# NETGEM: Network Embedded Temporal GEnerative Model for gene expression data

**DOI:** 10.1186/1471-2105-12-327

**Published:** 2011-08-08

**Authors:** Vinay Jethava, Chiranjib Bhattacharyya, Devdatt Dubhashi, Goutham N Vemuri

**Affiliations:** 1 Department of Computer Science and Engineering, Chalmers University of Technology, Göteborg, SWEDEN; 2 Computer Science and Automation Department, Indian Institute of Science, Bangalore, INDIA; 3 Systems Biology Division, Department of Chemical and Biological Engineering, Chalmers University of Technology, SWEDEN

## Abstract

**Background:**

Temporal analysis of gene expression data has been limited to identifying genes whose expression varies with time and/or correlation between genes that have similar temporal profiles. Often, the methods do not consider the underlying network constraints that connect the genes. It is becoming increasingly evident that interactions change substantially with time. Thus far, there is no systematic method to relate the temporal changes in gene expression to the dynamics of interactions between them. Information on interaction dynamics would open up possibilities for discovering new mechanisms of regulation by providing valuable insight into identifying time-sensitive interactions as well as permit studies on the effect of a genetic perturbation.

**Results:**

We present NETGEM, a tractable model rooted in Markov dynamics, for analyzing the dynamics of the interactions between proteins based on the dynamics of the expression changes of the genes that encode them. The model treats the interaction strengths as random variables which are modulated by suitable priors. This approach is necessitated by the extremely small sample size of the datasets, relative to the number of interactions. The model is amenable to a linear time algorithm for efficient inference. Using temporal gene expression data, NETGEM was successful in identifying (i) temporal interactions and determining their strength, (ii) functional categories of the actively interacting partners and (iii) dynamics of interactions in perturbed networks.

**Conclusions:**

NETGEM represents an optimal trade-off between model complexity and data requirement. It was able to deduce actively interacting genes and functional categories from temporal gene expression data. It permits inference by incorporating the information available in perturbed networks. Given that the inputs to NETGEM are only the network and the temporal variation of the nodes, this algorithm promises to have widespread applications, beyond biological systems.

The source code for NETGEM is available from https://github.com/vjethava/NETGEM

## Background

Gene expression microarrays are increasingly used to determine transcriptional regulation in response to a genetic or environmental perturbation. Often the inference is presented as a static network of genes that are activated or repressed by relevant transcription factors, similar to a wiring diagram of electrical circuits [1]. However, biological networks are inherently dynamic. In order to reveal the dynamics of the networks, substantial effort has been devoted to measuring the dynamics of gene expression or protein abundance. This information permitted identifying genes or proteins that substantially varied with time and their correlation to other cellular components, but not the interactions between cellular components. It is clear that dedicated mathematical models have to be generated to infer the dynamics of interactions in the biological networks.

Conventional methods of time series analysis cannot be applied to this problem due to the small number of observations (gene expression data) from different time points are available relative to variables (gene interaction strengths) [2]. Additionally, there is the inherent risk of many genes having similar expression profile, just by random chance. Recognizing these problems, it is only recently that dedicated methods are being developed to infer temporal regulation of transcription [3-5]. These, and other methods reviewed recently [6] do not consider the interaction networks connecting the genes nor any dependency of observations between time points and hence are not suitable for the problem at hand.

In this paper, we consider the problem of identifying temporal changes in the interactions in a network with a known topology from temporal profiles of gene expression data. The protein interaction network of baker's yeast, *Saccharomyces cerevisiae *is arguably the most well-constructed with a high level of confidence [7]. Therefore, we used this network to study and validate our models. The proteins in yeast are classified according to their biological function, as defined by MIPS [8]. This annotation scheme provides functional description of the proteins in a hierarchical structure to a high degree of resolution. This allows the possibility to relate functional classification of the network components with the temporal interactions between them. This line of reasoning leads to two very fundamental questions: (i) can we distill observations about temporal characteristics of a group of functionally similar genes? (ii) would it be possible to model the effect of a genetic perturbation (gene deletion or addition) while comparing temporal interactions between the reference strain and its perturbed mutant?

We introduce NETGEM, which stands for "Network Embedded Temporal GEnerative Model for gene expression data", a generative model for analyzing temporal data which is capable of capturing the interaction dynamics in the network. Our approach incorporates network effects into Markovian dynamics, and also compares the impact of genetic perturbation on the interaction dynamics. A fundamental premise of the model is that the evolution of the interaction strengths can be modeled in terms of the functional categories of the interacting genes. To the best of our knowledge, this is the first time such a model has been investigated. NETGEM assumes that the interaction strengths evolve *conditionally independent *of each other conditioned on the functional roles of the constituent genes. This assumption leads to a model where one can derive efficient inference procedures which have linear time complexity in the number of temporal observations. To handle the problem of low sample size, we adopt a Bayesian approach by introducing appropriate priors over the parameters governing the evolution of the interactions. Information from multiple mutants which differ from the reference strain in their network topology is incorporated by assuming that interactions closer to the perturbation (gene deletions) are affected more strongly than those further away.

Recent work [1,9,10] has focussed on learning locally sparse temporally rewiring interaction networks. Temporally rewiring gene networks were recovered by solving the intractable partition function evaluation using sampling techniques [9]. The key difference between our approach and the previous work is the generative model which encapsulates the functional category information. Further, the simplifications implemented in our work allow us to scale our inference procedure to large genetic networks.

Time-sensitive interactions were inferred from gene expression data using L1-regularization [10]. Such methods are extremely useful if the underlying network is not available. However, for the cases where the topology of the network is known, it is only logical to take advantage of this information and identify the interactions that change with time.

The main contribution of this paper is a generative probabilistic graphical model based on Markovian dynamics for temporal dynamics of gen e interactions that

• integrates known information about the underlying interaction network,

• encapsulates known functional category information to modulate the Markovian dynamics,

• develops a simple and fast method to jointly infer closely related models corresponding to small perturbations of a base network, and

• incorporates Bayesian approach to address the problem of small sample size.

Therefore, NETGEM takes the high throughput, temporal gene expression data and the network topology as inputs and scores the interactions based on the change in the expression value of the connected nodes over discrete time intervals. Furthermore, it has the built-in capability to assess the impact of a perturbation, such as a gene deletion, on the network. The underlying assumption is that the impact of the perturbation dampens with distance from the perturbed node. In order to provide physiological insight into the dynamics, the genes are classified according to their functional categories and the interaction between the functional categories is also assessed.

## Methods

### Datasets and Interaction Network

Dynamic gene expression datasets from *Saccharomyces cerevisiae *were downloaded from Gene Expression Omnibus using accession numbers GSE21988 and GSE9644. The first dataset contained the expression profiles of the genes in *S. cerevisiae *during the gradual increment in the availability of glucose. Therefore, the cells experience a transition from glucose starvation to nitrogen starvation (unpublished data). The data was measured at eight time points. The second dataset contained temporal gene expression profiles in *SFP1 *deletion mutant and its isogenic reference at six time points after pulsing steadily growing cells with glucose [11]. The reference strain is referred to as REF, the strain in which SFP1 was deleted is referred to as MUT. The raw data was obtained as CEL files, which was normalized and preprocessed in R programming environment using BioConductor suite of tools [12]. Background intensity in all the arrays was corrected using GeneChip Robust Multi-array Average algorithm [13]. The probe-level data was subjected to preprocessing by quantile normalization and median polish summarization.

The yeast interaction network was constructed using previously published data [14-19] as well as data downloaded from BIND [20], MIPS [8], MINT [21], DIP [22] and BioGRID [23]. We compiled all the interactions listed in these databases and retained only those interactions that were backed by at least two independent sources, resulting in a high-confidence protein interaction network. We excluded protein-DNA interactions since the result of this interaction is the gene expression and including these interactions would result in a cyclic relationship between the interactions and gene expression. We overlaid the gene expression data onto the protein interaction network. Therefore, inherent in this is the assumption that gene expression is translated into protein abundance uniformly among all proteins.

### Model description

We model the high confidence gene interactions network as a graph *G *= (*V, E*). We assume there are *S *perturbed networks or *strains*, indexed by the set {1, ..., *S*}, each having some genetic perturbation compared to the base network. Under different conditions, some of the edges are switched on or off, or, more generally set at various (discrete) levels of activation . Furthermore, an edge may be active in one strain and not in others at the same time.

Let  be a random variable on ℝ denoting the microarray gene expression level for gene *v *∈ *V *in strain *s *at time *t*, i.e. the event  indicates the microarray expression level of gene *v *in strain *s *at time *t *is . We note that the gene expressions levels are not restricted to a discrete set. Similarly, let  be a random variable on  representing the activation level of the edge *e *= (*i, j*) ∈ *E *between the genes *i *∈ *V *and *j *∈ *V *in strain *s *at time *t*.

We use the notation  and  to denote the gene microarray expression levels and the edge interaction strengths in strain *s *at time *t *respectively. For simplicity, we write  and  as  and  for all *e *∈*E *and *v *∈ *V *respectively. We begin by describing the overall proess dynamics as follows.

#### Observation model

We model the probability of the gene expression levels  conditioned on the interaction strengths  in the interaction network for strain *s *at time *t *as:(1)

where  is the normalization constant.

#### Evolution model

We assume that the weights evolve according to the Markov chain i.e.(2)

where  is the probability of the transition from state  at time *t *to state  at time (*t *+ 1) common for all strains {1, ..., *S*}. We assume that the overall dynamics for the interactions strengths in all the strains is governed by the same underlying factors, while instantaneous variations might occur in the interactions strengths due to local perturbations i.e.(3)

### Incorporating functional categories via mixtures

Since genes belong to multiple categories, a mixture model is a naturally suited model to handle the influence of multiple functional categories in the inference procedure. This allows us to explore the relationship between functional categories and the temporal evolution characteristics of the genes which fall in the same functional category. The remainder of this section describes the machinery for incorporating the functional category information into the generative model.

Let there be *H *possible gene functional categories, and each gene can be a member of one or more functional classes  where the hierarchical class *C_h _*is characterized by evolution matrix *Q_h_*. The evolution probability matrix *Q_e _*for each edge *e *∈ *E *is given as(4)(5)

Where *α_e,h _*denotes the influence of functional class *C_h _*in the edge *e *such that  for all edges *e *∈ *E *and *Q_e_*(*i, j*) denotes the (*i, j*) element of the edge transition probability matrix *Q_e_*.

#### Prior on transition probabilities (Θ)

The learning of the model parameters is significantly difficult when there are few time points. To alleviate this, we propose a prior Θ on the transition probabilities *Q_h_*. The individual rows  of the transition probability *Q_h _*can be thought about as drawn from a multinomial distribution. The Dirichlet distribution is the conjugate distribution [24] of the multinomial distribution and hence naturally suited as a prior distribution. We model the transition probabilities matrices as Dirichlet distributions, such that the prior on the transition probabilities matrix *Q *given the parameter Θ is(6)(7)

where  and  is the multinomial beta function [24]. Since, there is no information apriori about the functional categories, we chose a non-informative prior. Specifically. each row element of the prior is sampled from a uniform random distiribution; following which the row is normalized ().

#### Prior on mixing proportions (Λ)

We incorporate the effect of the functional classification of genes on the mixture components  for and edge e by using a Dirichlet prior of the form:(8)

with the prior parameter, *λ_e,h_*, for the edge, *e *= (*i, j*), of the form(9)

A value of *λ_p _= *1 and *λ_ο _*= 0 is used in the experiments.

#### Functional Category (Y )

We define the random variable  which denotes the functional category active at time *t *for edge *e*; such that the event  implies that .

### Analysis of Perturbed Networks

We consider the problem of multiple strains which are just slightly altered versions of the networks where a few genes have been knocked out of the network. Therefore, most of the network remains the same across strains with only the "close" neighbourhood of the knocked out genes being affected. We assume that the weights corresponding to the reference strain  evolve according to a Markov law given by a matrix *Q*, where  with the property that  for all the initial states **w***_l_*. For other strains, we assume that the corresponding values are just slightly perturbed; thus(10)

The perturbing parameters  are determined deterministically from the underlying network *G *by(11)

where  is a label determined by how far the gene *i *is in the underlying network to one of the genes knocked out in strain *s*. We note that the deterministic nature of the damping implies that all strains evolve similarly, i.e., *Q^s ^*= *Q*∀*s*. This allows us to incorporate the information for gene expression levels in the different strains while learning the temporal evolution characteristics.

We compute the damping factor  for the genes as follows: If the gene *i *is knocked out in strain *s *then we label it as . Now, we diffuse the labels across the graph such that  where *d*(*i*) and *N*(*i*) denotes the degree and the set of neighbors of gene *i *respectively, i.e., the damping factor at a node is the average of the damping factors at its neighbors. Here *β *is a hyper-parameter which can be used to control the damping effect. We investigated two schemes: a distance dependent *β *such that starting from the deleted node, all nodes at distance (in a breadth first search from the deleted node), greater than a selected distance have *β *= 0. We also investigated *β *in the range [0, 1]. Intuitively, while Γ*_e _*= 0 for an edge directly incident to one of the knocked out genes, the perturbation gradually damps out with distance from the knocked out gene and for an edge *e *far away from one of the knocked out genes Γ*_e _*≈ 1^. ^In the experiments, a value of  is used where *h_i _*denotes the distance of node *i *from the knocked-out node.

### NETGEM: a generative model

We now present a unifying view of the NETGEM model as a generative probabilistic model for gene expression data  for multiple strains *s *∈ {1, ..., *S*} and observation time points *t *∈ {1, ..., *T*}. The quantities known apriori are the number of classes *H*, the set of edges *E*, the number of strains *S *and the number of observation points *T*. The generative process corresponding to our model is given in Table 1.

**Table 1:** NETGEM Generative process

**Require: ***H *{set of functional categories}
**Require: ***G *= (*V, E*) {interaction network graph}
**Require: **Λ ={λ_e_}_*e*∈*E *_{prior on functional category mixtures}
**Require: **Θ ={*θ_h_*}_*h*∈*H *_{prior on class transition probability}
1: **for all ***h *in *H ***do**
2: Choose *Q_h_|θ_h _*s.t. each row
3: **for all ***e *in *E ***do**
4: Choose
5: **for ***t *= 1 to *T ***do**
6: **for all ***e *in *E ***do**
7: Choose
8: Choose where
9: Choose in (5)
10: Compute
11: Choose in (1)

Table 1 shows the generative process for the gene expression data  for multiple strains *s *∈ {1, ..., *S*} and observation time points *t *∈ {1, ..., *T*} in the NETGEM model. The active functional category  controls the change of interaction strength from  to  in edge *e *at time *t*. The interaction strengths affect the observed gene expression data, through the strain damping model and the conditional probability distribution in (1).

The key principle underlying the model is that the interaction dynamics are governed by the functional categories. In particular, for each edge *e *and time *t*, we generate a functional category . This functional category is *solely *responsible for the change of interaction strength from  to . The interaction strengths affect the observed gene expression data, through the strain damping model and the conditional probability distribution in (1). On the whole, this is equivalent to a mixture of the corresponding functional categories with appropriate mixing proportions.

### Inference

The hidden variables are  and the parameters to be learnt are Ψ = {*Q_h_*, *α*_*e*,*h*_}. The hyper-parameters Λ and Θ are chosen as non-informative priors. An Expectation Maximization (EM) [25] procedure is used to obtain the iterative update equations for  and . Details on choice of hyper-parameters and E.M. procedure are provided in Additional File 1 accompanying this paper.

### Selection of significant interactions

The E.M. inference procedure in our model estimates the most probable sequence of states  for all edges *e *∈ *E *over the simulation time period. However, many edges have few changes, and we need to select the interactions having temporally significant interactions based on a suitable metric.

The dynamics of the interaction strength  of an edge *e *is governed by the Markov transition probability *Q_e _*in our model. Therefore, we characterize the evolution of interaction strengths as significant or not significant under the hypothesis [26]

*H*0 : The interaction *w_e_*(*t*) on an edge *e *∈ *E *is not significant if the transition probability matrix *Q_e _*has more than *P *% of its mass on the diagonal, i.e. ,

*H*1 : The interactions *w_e_*(*t*) on an edge *e *∈ *E *is significant if the transition probability matrix *Q_e _*has at most *P *% of its mass on the diagonal, i.e. ,

where *tr*(*Q_e_*) denotes the trace of Markov transition probability *Q_e _*for edge *e *given as(12)

and  denotes the total number of possible interaction strengths for an edge in the network.

The choice of an appropriate test statistic is a non-trivial matter. For example, the weights {-2, -1, 0, 1, 2} on an edge *e *= (*g, g*') corresponds to increasing degree of positive correlation between the expression data for genes *g *and *g*'. Therefore, a change from *w *= -2 *(strongly repressing) *to *w *= +2 *(strongly activating) *is more significant than a change from *w *= -2 to 0 *(uncorrelated)*. Further, the transition probabilities for the edges *Q_e _*are dependent on the functional categories transition probabilities *Q_h_*.

We use the test statistic *s_T _*(*e*) which measures the degree of change exhibited by the edge (interaction), *e*;(13)

where *T *is the total number of observations and *w_e_*(*t*) is the interaction strength of edge *e *at time *t*. We define change score  for the functional category *C_h _*as(14)

The decision rule based on *s_T _*(*·*) is given as:

*d*0 : The interaction for edge *e *(functional category *h*) does not have temporally significant dynamics if , and

*d*1 : The interaction for edge *e *(functional category *h*) has temporally significant dynamics if ,

where s* is the critical value.

The test statistic *s_T _*(*e*) is a random quantity depending on the Markov transition probability *Q_e _*describing the evolution of interaction strengths *w_e_*(*t*) defined on edge *e*. However, the change score *s_T_*(*e*) generally decreases with increasing trace *tr*(*Q_e_*). Please see the supplementary material (Additional File 1) accompanying this manuscript for discussion on change score.

**Figure 1:**
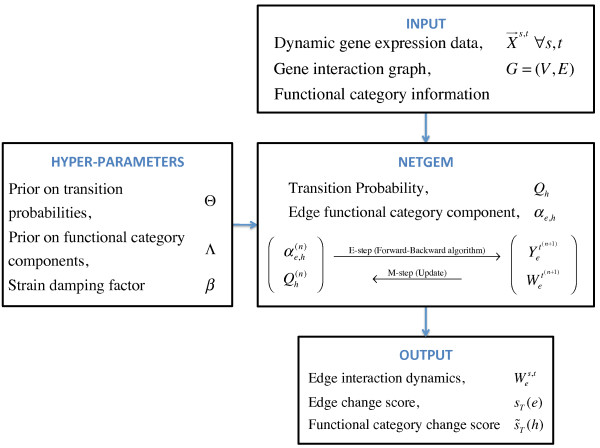
**The NETGEM model**. This figure presents a high-level description of the NETGEM model and the resulting inference algorithm. Here Λ, Θ are hyper-parameters and *β *is the strain damping factor. The parameters to be learnt are the functional category component for each edge (α*_e_*) and the functional category transition probability matrix (*Q_h_*). The inference is done over the hidden variables , where  and  are the random variables corresponding to the active functional category and the interaction strength for edge *e *at time *t *respectively. The inferred interaction dynamics  is used to compute the edge and functional category change scores *s_T _*(*e*) and  respectively.

Figure 1 presents a high-level description of the NETGEM generative model and the resulting inference algorithm. The hyper-parameters Θ and Λ and the strain damping factor *β *are specified by the user. The inference is done over the hidden variables ; and the parameters to be learnt are Ψ = {*Q_h_*, *α*_*e*,*h*_}. The inferred interaction dynamics  is used to compute the edge and functional category change scores *s_T _*(*e*) and  respectively.

## Results and Discussion

We first analyzed the performance of our model *NETGEM *(Figure 1) using synthetic data to investigate the impact of functional categories on the inference.

### Evaluation of NETGEM with synthetic data

We construct a synthetic graph *G *= (*V*, *E*) using the Erdos-Renyi model consisting of *|V | *= 1000 nodes, which represents the genes, and |*E*| = 5961 randomly generated edges, which represent the gene interactions. Each edge *e *∈ *E *can have one of the following states , signifying the interaction strength. We consider two models for interaction dynamics in the genetic network to investigate the impact of functional category information on the interaction dynamics in the gene interaction network, which are described below:

• The first model incorporates the functional categories, which impact the evolution of interaction strengths as specified in eqn. (4) and eqn. (5). We use *H *= 200 functional categories which govern the behaviour of the evolution of the gene expression. Nodes were randomly assigned to (multiple) functional categories reflecting the empirical distribution of functional categories for genes in MIPS database [8] (See section D in Additional File 1 for details). The transition probability matrix for the functional categories *Q_h _*were sampled randomly from two classes *H*0 : *tr*(*Q_h_*) *>*0.5*W *and *H*1 : *tr*(*Q_h_*) *≤ *0.5*W*. The interaction dynamics for each edge (*Q_e_*) depends on the functional categories (in terms of *Q_h_*) that influence the nodes of the edge as in eqn. (4).

• The second model assumes the evolution of the interaction strengths for an edge is independent of other edges. The transition probability matrix *Q_e _*for each edge *e *∈ *E *is generated by sampling with equal probability from the two classes *H*0 : *tr*(*Q_e_*) *>*0.5*W *and *H*1 : *tr*(*Q_e_*) *≤ *0.5*W*.

We simulate the dynamics for interactions for *t *∈ {1, ..., 8} for each of the two models by generating interaction strengths  for each edge *e *by sampling from a Markov chain with random starting state and transition probability *Q_e _*chosen according to the model. At each time instant, we generate observation  at each node *x *∈ *V *in {-1, 1} based on the interactions  at time *t *as in eqn. (1) using Gibbs sampling [27]. This artificial data is analogous to gene expression data in our model. The simulation period chosen (*T *= 8) is small for generic statistical inference techniques, reflecting the size of gene expression datasets commonly available [4,28].

The inference in our model is done to learn the evolution dynamics of the functional categories based on the interactions of multiple genes which have the corresponding functional classification. Based on the inferred interactions, we compute the change score *s_T _*(*e*) and  for each edge *e *and functional category *h *as in eqn. (13) and eqn. (14) respectively. The change score allows us to classify the edge (or functional category) as belonging to class *H*0 or *H*1 based on the choice of critical score s*. If we chose the critical value s* to be very high, most of the edges or functional categories will be classified as insignificant (belonging to class *H*0), as their test statistics *s_T _*(*e*) or  will be less than the critical value s*. Conversely, if we chose the critical value s* to be 0, most of the edges or functional categories will be classified as significant (belonging to class *H*1), as their test statistics *s_T _*(*e*) or  will be more than the critical value s*.

**Figure 2:**
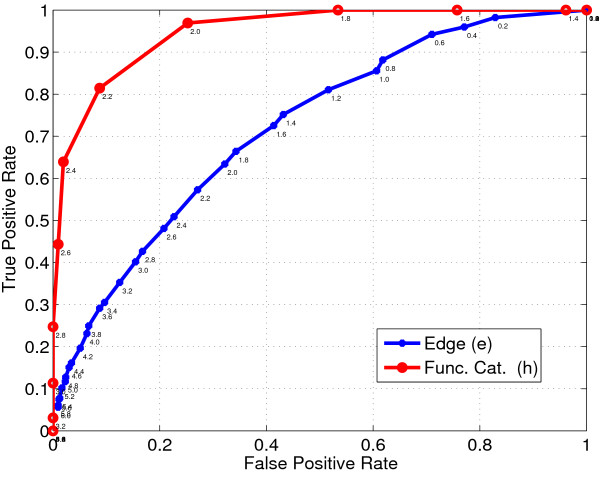
**R.O.C. comparison of functional categories and edges**. This figure compares the ROC plots for detecting significant edges *e *and functional categories *C_h _*based on corresponding test statistic *s_T _*(*e*) and . The experiment was conducted for *T *= 8 simulation period. The critical value of the test statistic is indicated next to the plots.

We characterize the sensitivity of the test statistic using the Receiver Operating Characteristics (ROC) curve based on different critical values of the test statistic s*. Figure 2 shows the comparison in ROC plots of the edges *e *∈ *E *and functional categories *h *∈ *H*, based on the corresponding test statistics *s_T _*(*e*) and  in eqn. (13) and eqn. (14) respectively. The critical values of the test statistic s* are indicated next to the obtained operating characteristics. The Area Under Curve (AUC) values for functional categories and edges are 0.9484 and 0.7208 respectively, which reflects the increased accuracy in determining the dynamic functional categories compared to edges. We note that the multiple edges corresponding to a functional category improves the detection of significant functional categories even under short time periods. If the gene expressions were truly independent of the function of the genes, one would observe fairly low accuracy in predicting the most dynamic functional categories. Additional File 1 accompanying this manuscript presents additional experiments discussing the choice of change score as a test statistic for selection of significant interactions.

Further, the strain damping allows the incorporation of multiple gene expression datasets which have been generated under slightly different conditions. In effect, we learn the dynamics of functional categories from multiple instances of short time series which are not i.i.d. but are strongly related through the functional classification. This reduces the variance in the results partly alleviating the problem of inference in short time series.

One can model the dynamics of the simple model defined by eqn. (1) and eqn. (2) using a simple HMM having a large state space. The quantity to be estimated is the transition probability **Q**. However, the exponential state space makes such an approach impractical. For the purpose of study, we present a small experiment which shows that incorporating the functional information in beneficial in terms of computational requirements as well as accuracy (See Additional File 1 for details).

### Using NETGEM on gene expression data

This section presents inference results using our model for identifying interaction dynamics from two gene expression datasets in yeast.

We are interested in the edges which show considerable activity during the measured time points. Towards this end, we compute the change score *s*(*e*) as in eqn. (13). We fit an exponential distribution to it and consider the weights falling in the top-5% (*p *= 0.05) tail of the distribution. The interaction between the nodes was visualized as a graph in the Cytoscape [29] environment.

Our results show that the model correctly identifies known interactions. For example, it discovers the gradual transition from positive to negative interaction strength in edges between carbohydrate metabolism and protein synthesis genes. Moreover, it detects abrupt changes in the interaction patterns. Combining expression data from a reference strain and *Sfp1 *knockout strain shows that our model is able to successfully integrate multiple strains via strain damping. It also implicates many new actively interacting genes which have an important role in the biological conditions corresponding to the gene expression datasets. These can be used as test candidates in future biological experiments.

The gene expression dataset in the following experiments is normalized independently for each strain by subtracting from the gene expression  at node *v *at time *t *in strain *s *as(15)

where  denotes the mean gene expression level in strain s.

We chose the set  as the set of possible interactions for each edge. An (inferred) interaction of *w_e _*= +2, *w_e _*= -2 and *w_e _*= 0 means the gene expressions for the genes of edge *e *is strongly positively correlated, strongly negatively correlated or uncorrelated respectively. Similarly, an interaction of *w_e _*= +1 or *w_e _*= -1 indicates weak positive or negative correlation between the expressions for the genes of the edge *e *respectively. Our choice of  allows us to infer the degree of the correlation in addition to its sign. If this information is not required, one could choose a smaller state space (e.g. {-1, 0, 1}) which would reduce the overall complexity. Alternatively, if finer inference is required, one can choose a larger state space at the cost of higher computational complexity and additional prior information that has to be provided.

#### Interaction dynamics in response to nutrient availability (Experiment 1)

The data in this experiment captures the changes in gene expression during the gradual transition from glucose to ammonia as the growth-limiting nutrient. Genes that have already been grouped into eight clusters [11] were connected according to the protein interaction network and the interaction strength between them was determined from gene expression using the model. At the beginning of the experiment (t = 0 h), the cells were starved for glucose and were progressively exposed to increased glucose availability. After time t = 9.6 h, ammonia became the limiting nutrient. Subsequent time points capture the changes that occur in the presence of excess glucose. In response to this transition, we observed corresponding changes in glucose and ammonia metabolism, which are reflected in the interactions between the genes responsible for the synthesis of proteins and lipids.

**Figure 3:**
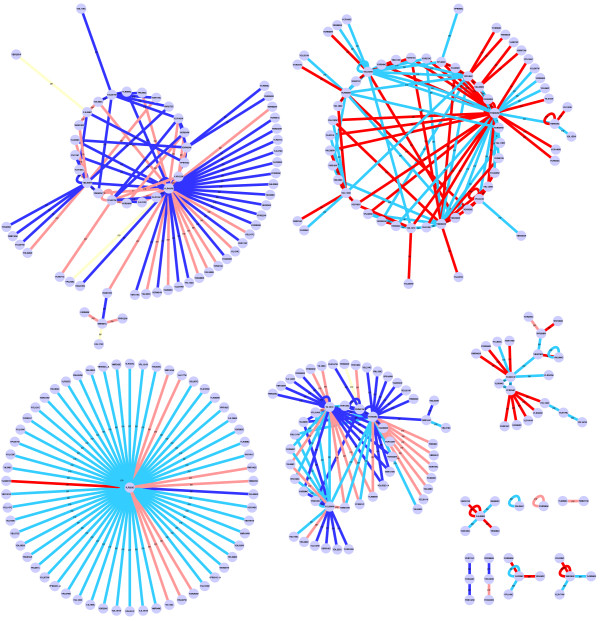
**Experiment 1 network interactions**. Time varying interaction strengths between the genes from Experiment 1. Each network is composed of all the genes from the eight clusters previously identified [11], and is shown for *t *= 0.0 hrs. The edge colors denote their interaction strength, which was classified as strong repressing (red), low repressing (pink), no effect (yellow), low inducing (light blue) and strong inducing (dark blue).

Figures 3 shows the inferred interactions at *t *= 0.0 h. Please see the supplementary material (Additional File 1) accompanying this manuscript for the inferred dynamics for all eight time points, as well as the list of twenty most dynamic gene-gene interactions. Our model identified strong positive (inductive) interaction between the genes of carbohydrate metabolism and protein synthesis (top left corner of the network) during glucose starvation. The interaction strength gradually decreased until t = 9.6 hours and subsequently turned negative (repressive). Since the genes in this cluster predominantly belong to amino acid synthesis, our results indicate a gradual repression in amino acid and protein synthesis upon the onset of ammonia starvation.

The transition in the growth-limiting nutrient also brought about an abrupt change in the interactions between genes responsible for ribosome biosynthesis (cluster of genes on the extreme right of each network). Our model identified a momentary repression in the synthesis of ribosomes at t = 9.6 hours, when the growth limitation was exerted by ammonia. These genes were constitutively active during all other time points, as is expected because ribosome biosynthesis is an essential cellular process. The temporary arrest in ribosome biosynthesis was attributed to the control exerted by Sfp1 transcription factor [11]. Our model also identified positive interactions between amino acid metabolism and cell cycle and negative interactions between genes of storage carbohydrates and lipid metabolism.

#### Interaction dynamics in response to network perturbation (Experiment 2)

An important aspect of NETGEM is to capture the dynamics in response to a perturbation in the network. The model allows identifying the significantly changed interactions in response to a deletion of a gene. In this experiment, we evaluate the effect of deleting a key transcription factor, Sfp1 [30]. We chose this dataset since Sfp1 was previously identified to be one of the most important transcription factors that governed the response to nutrient availability in yeast [11].

We first identified the interactions that are sensitive to time and perturbation. The histogram of the number of edges was fit to an exponential distribution and only those interactions that passed the threshold cutoff of *p *< = 0.05 were considered for subsequent analysis. In this manner, we identified 171 interactions among the genes that were already identified to have been differentially expressed between REF and MUT. Please see the supplementary material (Additional File 1) accompanying this manuscript for details.

An important aspect of novelty in our model is the incorporation of the damping effect in the model through eqn. (10) and eqn. (11). This ensures that interactions further from the point of perturbation in the network are affected to a lesser degree than those closer to it. The effect of damping is very sensitive to the network and in the network we considered in this study, a majority of the edges appear to be relatively unaffected by the perturbation.

**Figure 4:**
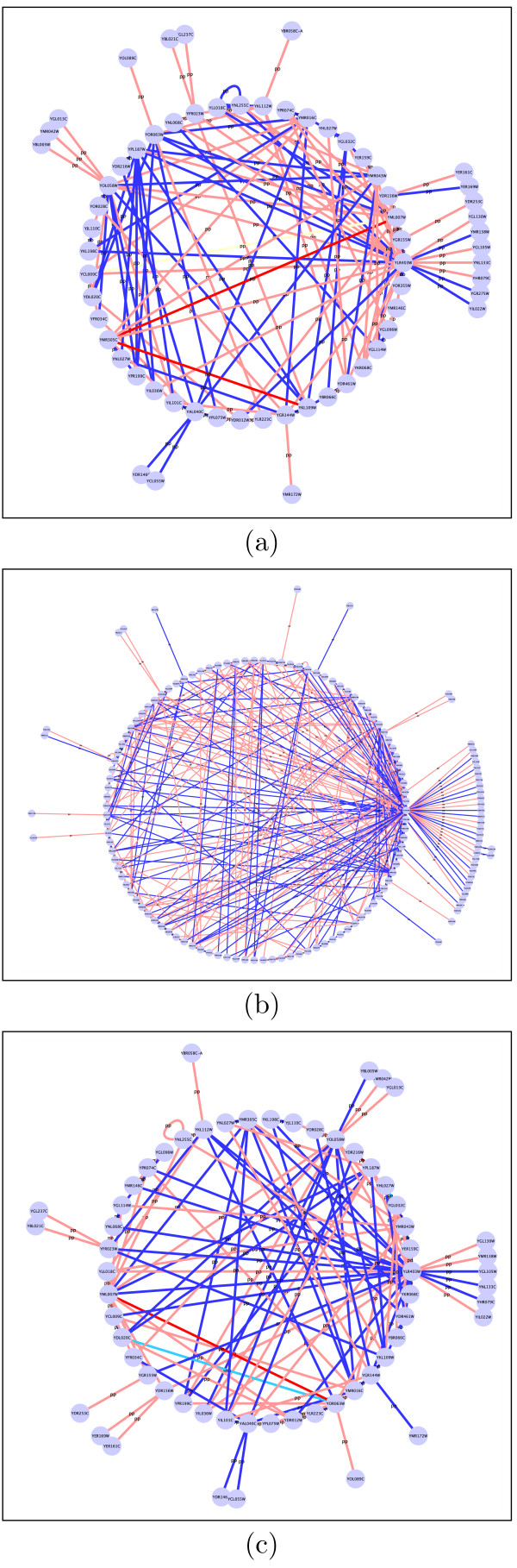
**Experiment 2 network interactions**. Dynamics of temporal interactions between genes in (*a*) REF, (*b*) MUT and in (*c*) both strains combined, using the damping model for Experiment 2 at *t *= 0 min. All the genes identified to be significantly changed [30] were combined into one network. The color of the edges in the network indicates the interaction strength, which was classified as strong repressing (red), low repressing (pink), no effect (yellow), low inducing (light blue) and strong inducing (dark blue).

After assessing the effect of perturbation for our network, we identified temporal changes in the interactions in the REF strain as well as the MUT strain independently. We observed some overlap in the actively interacting genes between REF and MUT. Many of these genes were hexose transporters and those responsible for pH homeostasis. The genes (and the functional categories) that are common to the strain indicate that they are not responsive to the mutations. NETGEM was able to identify temporal interactions that are most sensitive to the mutation. These interactions predominantly occurred between ribosome biosynthesis and amino acid metabolism. The results concur with the known role of Sfp1 in coordinating metabolism with ribosome biosynthesis and serve as an independent validation of the accuracy of the damping model incorporated in NETGEM. These interactions were identified by considering gene expression profiles in REF and MUT, using the damping model. Indeed the functional classification of the genes between which interactions change significantly indicate that Sfp1 transcription factor has widespread control over coordinating ribosome biosynthesis, pH homeostasis, transport of proteins and drugs, etc. Table 2 and Table 3 show the list of MIPS IDs corresponding to the 20 topmost functional categories whose transitional probabilities exhibit the maximum degree of change in interaction strengths out of 260 possible functional categories, and the description of the functional categories corresponding to the MIPS IDs respectively. This is obtained by considering the total probability of change in the transition probability matrix, *Q_h_*, i.e., . The fact that many of these functional categories have already been identified [30] to be sensitive to the perturbation gives substantial credibility to our findings. Figure 4 presents the temporal dynamics of the interaction strengths in (*a*) REF, (*b*) MUT, and (*c*) JOINT where the inference is based on both the strains combined to *t *= 0 min.

**Table 2:** Functional categories with maximum temporal variation

REF	MUT	JOINT
**32.01.04**	**32.01.04**	**32.01.04**
**20.09.03**	**20.09.03**	**20.09.03**
10	16.21	16.21
16.21	32.05.05	**11.04.02**
**11.04.02**	10	10
32.05.05	**11.04.02**	**12**
**20.01.27**	40.01.05	32.05.05
**30**	**30**	**30**
16.03.03	10.01.09	16.03.03
**20.09.07**	10.03.01	32.07
**12**	14.13.01	**20.09.07**
32.07	**20.01.10**	40.01.03
**20.01.15**	16.03.03	**20.01.27**
42.04.05	**20.09.07**	10.01.09
34.07.02	42.04.03	34.07.02
10.03.01	16	32.07.07
40.01.03	34.07.02	16.02
10.01.09	**12.01.01**	42.04.05
42.29	**01.03.01**	42.29
32.07.07	32.07.07	14.04

Table 2 shows the MIPS IDs of the top 20 functional categories which show the maximum amount of variation in time for REF or MUT strains, evaluated independently and Jointly using the damping model. This is obtained by considering the total probability of change in the transition probability matrix *Q_h _*i.e. . The categories indicated in bold font are those which are known to have been enriched in the original dataset [30].

**Table 3:** Description of functional categories

MIPS ID	Description of functional categories
**01.03.01**	purin nucleotide/nucleoside/nucleobase metabolism
10	cell cycle and DNA processing
10.01.09	DNA restriction or modification
10.03.01	mitotic cell cycle and cell cycle control
**11.04.02**	tRNA processing
**12**	protein synthesis
**12.01.01**	ribosomal proteins
14.04	protein targeting, sorting and translocation
14.13.01	cytoplasmic and nuclear protein degradation
16	protein with binding function or cofactor requirement (structural or catalytic)
16.02	peptide binding
16.03.03	RNA binding
16.21	complex cofactor/cosubstrate/vitamine Binding
**20.01.10**	protein transport
**20.01.15**	electron transport
**20.01.27**	drug/toxin transport
**20.09.03**	peroxisomal transport
**20.09.07**	vesicular transport (Golgi network, etc.)
**30**	cellular communication/signal transduction mechanism
**32.01.04**	pH stress response
32.05.05	virulence, disease factors
32.07	detoxification
32.07.07	oxygen and radical detoxification
34.07.02	cell-matrix adhesion
40.01.03	directional cell growth (morphogenesis)
40.01.05	growth regulators/regulation of cell size
42.04.03	actin cytoskeleton
42.04.05	microtubule cytoskeleton
42.29	bud/growth tip

Table 3 presents the description of the functional categories corresponding to the MIPS IDs present in Table 2.

Please see Additional File 2 for the Cytoscape attribute files and the figures corresponding to Experiment 1 and Experiment 2.

## Conclusion

There is a trade off between using more sophisticated conditional probability models *p*(**w***^s^*(*t*)*|***w**^0^(*t*)) involving more parameters to be learnt and the limited amount of experimental data in order to model interaction dynamics. NETGEM is a systematic model that relates temporal changes in gene expression data to the dynamics of interactions in the context of a regulatory network. We believe that NETGEM achieves an optimal balance between model complexity and the data requirement, while allowing ample flexibility to adjust the parameters. The framework of the model will also inherently facilitate analyzing the effect of a perturbation in the network. For a given regulatory network and gene expression data, NETGEM was able to identify time-sensitive interactions in the network and determine their strength. It was able to deduce the most active functional categories that interacted. In addition to these, the NETGEM uses a damping feature that models the effect of a network perturbation by localizing more activity around the point of perturbation. These three novel features that NETGEM offers reflect its advantage over many other time-series models that have been developed recently. Of particular interest is its ability to capture abrupt changes in the interaction patterns. For example, NETGEM identified momentary arrest in ribosome biosynthesis during the transition in the nutrient that limits growth from glucose to ammonia (Experiment 1). We identified many actively interacting genes that were implicated to play an important role in the biological conditions from which we obtained the data. This lends the promise that new insights obtained from using NETGEM are also physiologically relevant. Given that the inputs to NETGEM are the topology of the network and temporal variation of the nodes, it is evident that this methodology has widespread applications in analyzing network dynamics, beyond biological systems.

## Authors' contributions

VJ, CB, DD and GNV designed the study. GNV provided the experimental data and critically revised the biological findings of the method. VJ performed the analysis. All authors interpreted the results. VJ and GNV wrote the manuscript. All authors read, edited and approved the final manuscript.

## Supplementary Material

Additional file 1 **Supplementary material**. This file contains the supplementary material accompanying this manuscript. Click here for file

Additional file 2 **Cytoscape visualization data**. This file is a zip archive containing the Cytoscape attribute files and the figures corresponding to Experiment 1 and Experiment 2. See the included README file for more details. Click here for file
