# Supplementary material for: NETGEM: Network Embedded Temporal GEnerative Model for gene expression data
Source: BMC Bioinformatics. 2011 Aug 8;12:327. doi: 10.1186/1471-2105-12-327 (PMC3228555; doi:10.1186/1471-2105-12-327)
Supplement: Additional file 1 — Supplementary material. This file contains the supplementary material accompanying this manuscript. [file 1471-2105-12-327-S1.PDF]

Supplementary material for manuscript: "NETGEM: Network embedded temporal generative model for gene expression data"

Contents

A Model derivation **2**

B Prior Initialization **3**

C Empirical properties of change score $s(e)$ **4**
- C.1 Impact of number of observations 4
- C.2 Empirical distribution of $s(e)$ 5
- C.3 Test statistic sensitivity 5

D Impact of small number of observations **6**

E Discussion on NETGEM vs Naive HMM **9**
- E.1 Evaluation of independent weights dynamics 9
- E.2 Number of parameters to be learnt 10
- E.3 Experimental comparison 10

F Experiment 1 results **11**

G Experiment 2 results **13**

This document presents the supplemental material for the manuscript "NETGEM: Network Embedded analysis of Temporal Gene Expression Model".

A Model derivation

This section presents the derivation for the inference and parameter learning in the generative model, NETGEM. The inference is done over the hidden variables $\{y_e(t), w_e(t)\}$ based on the Markovian structure of the problem. The expectation maximization procedure Neal and Hinton [1998] which iteratively learns the parameters $\Psi = \{Q_h, \alpha_{e,h}\}_{h\in\mathcal{H},e\in E}$ conditioned on hidden variable $\Omega = \{\mathbf{y}^{1:T}, \mathbf{w}^{1:T}\}$ as

$$\begin{array}{llll} \text{E-step:} & \mathcal{L}(\Psi;\Psi^{(n)}) & = & E_{\Omega^|}[\ln P(\mathbf{x}^{1:S}(1:T), \Omega(1:T)|\Psi(1:T))] \\ \text{M-step:} & \hat{\Psi}^{(n+1)} & = & \arg\max_{\Psi}(\ln P(\Psi) + \mathcal{L}(\Psi;\Psi^{(n)})) \end{array} \quad \text{(A.1)}$$

where $\Omega^|$ is the conditioned variable $(\mathbf{w}^{1:T}, \mathbf{y}^{1:T}|\mathbf{x}^{1:S}(1:T), \Psi^{(n)})$.

We assume that the data for strain, s, is independently generated based on the Ising model with the weights that are damped versions of the weights in the original strain. This leads to the observation model specified as:

$$o_e^t(l) = P(x_e^{1:S}(t)|w_e(t) = w_l) \quad \text{(A.2)}$$

$$= \frac{1}{Z}\prod_{s=1}^{S} P(x_e^s(t)|w_e(t) = w_l) \quad \text{(A.3)}$$

$$= \frac{\exp\left\{-w_l(\sum_{s=1}^{S} x_i^s(t)x_j^s(t)\Gamma^s(i,j))\right\}}{\sum_{l=1}^{\mathcal{W}}\exp\left\{-w_l(\sum_{s=1}^{S} x_i^s(t)x_j^s(t)\Gamma^s(i,j))\right\}} \quad \text{(A.4)}$$

The forward iterates, $f_e^t(l,h)$ and backward iterates, $b_e^t(l,h)$ can be computed as follows:

$$f_e^t(m,h) = P(x_e^{1:S}(1:t), w_e^t = w_m, y_e^t = h|\Psi_e^{(n)}) \quad \text{(A.5)}$$

$$= P(x_e^{1:S}(t)|w_m)\sum_{w_l}\sum_{h'}\Big[P(y_e^t = h|\alpha^{(n)})$$

$$\times\ P(w_m|w_e^{t-1} = w_l, y_e^{t-1} = h') \times f_e^{t-1}(l,h')\Big] \quad \text{(A.6)}$$

$$= o_e^t(m)\sum_{l=1}^{M}\sum_{h'=1}^{H} f_e^{t-1}(l,h')\alpha_h^{(n)}q_{h'}^{(n)}(l,m) \quad \text{(A.7)}$$

$$b_e^t(m,h) = P(x_e^{1:S}((t+1):T)|w_e(t) = w_m, y_e^t = h, \Psi_e^{(n)}) \quad \text{(A.8)}$$

$$= \sum_{w_l}\sum_{h'}\Big[P(x_e^{1:S}(t+1)|w_e^{t+1} = w_l)b_e^{t+1}(l,h')$$

$$\times\ P(w_e^{t+1} = w_l|w_m, y_e^t = h)P(y_e^{t+1} = h'|\alpha^{(n)})\Big] \quad \text{(A.9)}$$

$$= \sum_{l=1}^{M} \sum_{h'=1}^{H} q_h^{(n)}(m,l) o_e^{t+1}(l) \alpha_{h'}^{(n)} b_e^{t+1}(l,h') \quad \text{(A.10)}$$

where $M = |\mathcal{W}|$ denotes the number of possible states of the interactions on each edge. The conditional probability $P(\Omega_e^t = (w_l, h), \Omega_e^{t+1} = (w_m, h') | \mathbf{x}_e^{1:S}(1 : T), \Psi^{(n)})$ denoted by $\xi_e^t(l,m,h,h')$ can be computed as

$$\xi_e^t(l,m,h,h') \propto f_e^t(l,h) \alpha_{h'}^{(n)} q_h^{(n)}(l,m) o_e^{t+1}(m) b_e^{t+1}(m,h') \quad \text{(A.11)}$$

The likelihood term, $\mathcal{L}(\Psi; \Psi^{(n)})$ in (A.1) can be expressed in terms of the conditioned edge probabilities, ξ_e^t, in (A.11) as

$$\mathcal{L}(\Psi; \Psi^{(n)}) = \sum_{e \in E} \sum_{t=1}^{T-1} \mathbf{E}_{\xi_e^t} [\ln q_h(l,m) + \ln \alpha_{e,h'}] \quad \text{(A.12)}$$

subject to the constraints

$$\sum_m q_h(l,m) = 1 \quad \forall h \quad \text{(A.13)}$$

$$\sum_h \alpha_{e,h} = 1 \quad \forall e \quad \text{(A.14)}$$

B Prior Initialization

The priors in the generative model play a crucial part in the model. We briefly discuss them below:

- θ: This is the prior over the transition probability matrices for the functional classes. Since, there is no information apriori about the functional categories, we chose a non-informative prior. Specifically. each row element of the prior is sampled from a uniform random distiribution; following which the row is normalized.

- λ: This is the prior over the mixing proportions and is used to encapsulate the functional category information. The matrix is initialized as $\alpha_{e,h} = 0$. For each functional category h that is known for a gene of an interacting edge, e, a value of 1 is added in the corresponding $\alpha_{e,h}$. A gaussian noise with $\sigma \sim 0.1$ is introduced; following which the rows are normalized such that $\sum_h \alpha_{e,h} = 1 \forall e$.

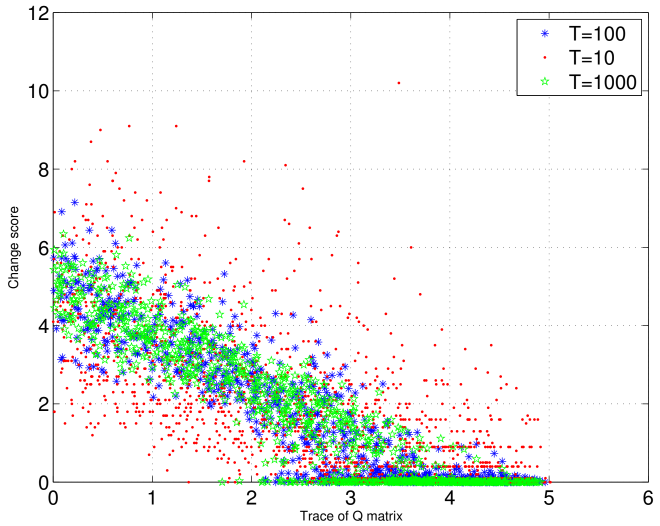

Figure 1: This figure shows the dependence between trace of the Markov transition probability matrix, $tr(Q_e)$, and the score, $s_T(e)$, at different simulation periods, $T = 10$, $T = 100$, $T = 1000$.

C Empirical properties of change score $s(e)$

This section presents experiments using artificial data to study the properties of the change score as test statistic.

C.1 Impact of number of observations

Figure 1 shows the dependence of the change score $s_T(e)$ on the trace $tr(Q_e)$ of the Markov transition probability matrix Q_e and different simulation time periods, T. We observe that the change score $s_T(e)$ decreases with increasing trace $tr(Q_e)$ even at small simulation time period ($T = 10$). Further, the variance in the scores for a given trace $tr(Q_e)$ decreases with larger values of simulation period T. However, at small observation periods $T \leq 10$, there is a significant degree of randomness in the change scores. This is indeed the case for any short time series data, which follows some underlying distribution.

In the remainder of this section, we focus on the case when the number of observations is small $T = 8$. We denote the change score by $s(e)$ for this case.

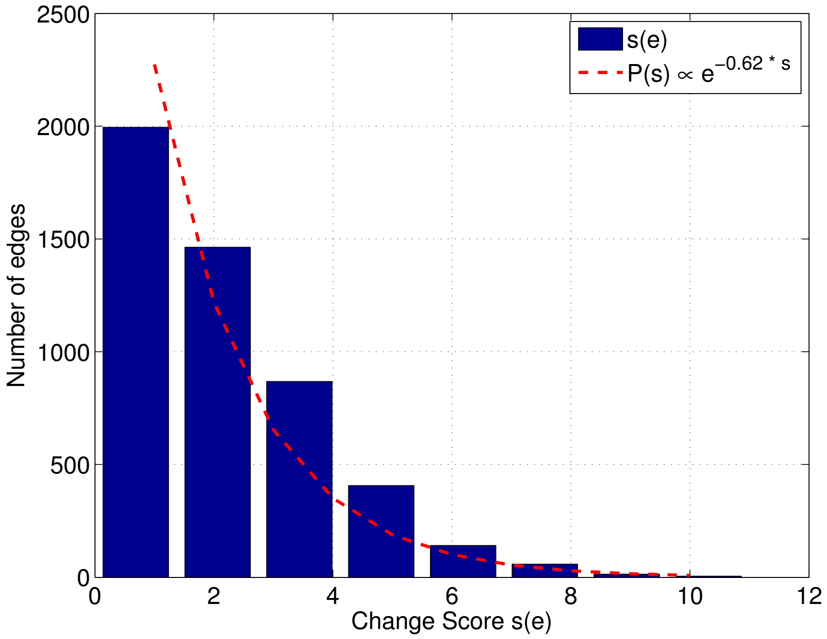

Figure 2: The histogram of the change scores for synthetic experiment and the best exponential fitted to the data.

C.2 Empirical distribution of $s(e)$

The underlying distribution of the test statistic $s(e)$ is unknown. However, based on empirical data, we assume that the distribution of the test statistic $s_T(e)$ has an exponential distribution.

Figure 2 shows the empirical distribution of the change scores $s(e)$ for a simulation on a graph consisting of 1000 nodes and 5961 edges, and the best exponential distribution fitted to the empirical data. Figures 3 (a) and (b) show the empirical distribution of the test statistic under the null $(H0 : tr(Q_e) > 0.5W)$ and alternate hypothesis $(H1 : tr(Q_e) \leq 0.5W)$.

C.3 Test statistic sensitivity

We characterize the sensitivity of the test statistic using the Receiver Operating Characteristics (ROC) curve. Figure 4 (a) shows the Receiver Operating Characteristics (ROC) plots for the synthetic experiment under the null hypothesis $H0 : tr(Q_e) > 0.5W$ and the alternate hypothesis $H1 : tr(Q_e) \leq 0.5W$. The critical value of the test statistic s^* are indicated next to the

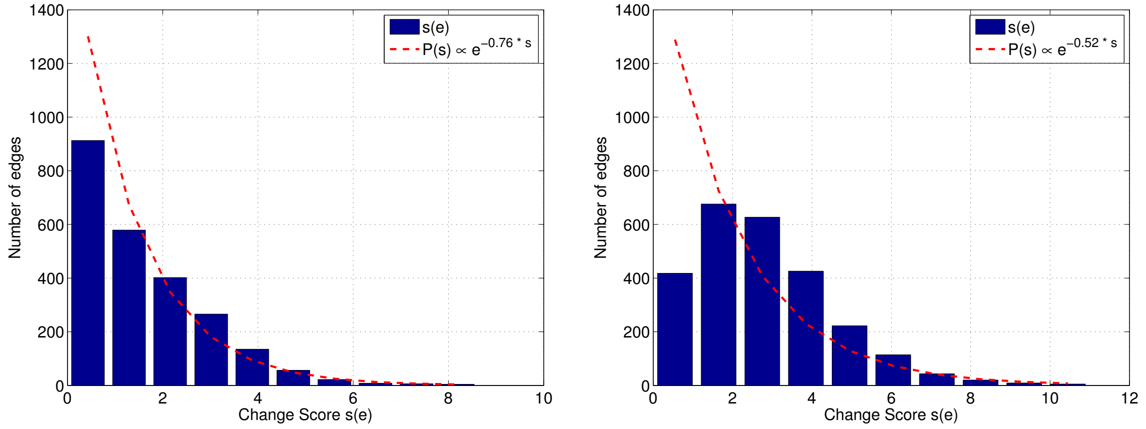

$(a)\ H0$ $(b)\ H1$

Figure 3: Figure (a) and (b) shows the empirical distribution (blue) of the test statistic under the null ($H0$) and alternate hypothesis ($H1$) respectively. The test statistic distribution is approximated using the best exponential fit (red).

obtained operating characteristics. We note that the increasing value of the critical value s^* reduces the number of false positives.

Figure 4 (b) shows the distribution of the p-values under the null hypothesis.

D Impact of small number of observations

This section discusses the impact of the small number of noisy observations (gene expression data). While it is desirable to have gene expression at sufficiently large number of data points to warrant satisfactory application of the conventional time-series analyses to glean useful information, such an experimental setup is often prohibited by expenses and time. Therefore, it is common to extract biological information by measuring gene expression at fewer time points, usually in the range of $5 - 10$ Ernst and Joseph [2006], Ernst et al. [2005]. It is to be noted that the rate of change in the expression of the genes is the ultimate determinant of the number of measurements. For example, too few measurements in a rapidly changing system would fail to capture crucial dynamics, while a large number of measurements on a slowly changing system would obscure reliable inferences.

The inference in NETGEM is done to learn the evolution dynamics of

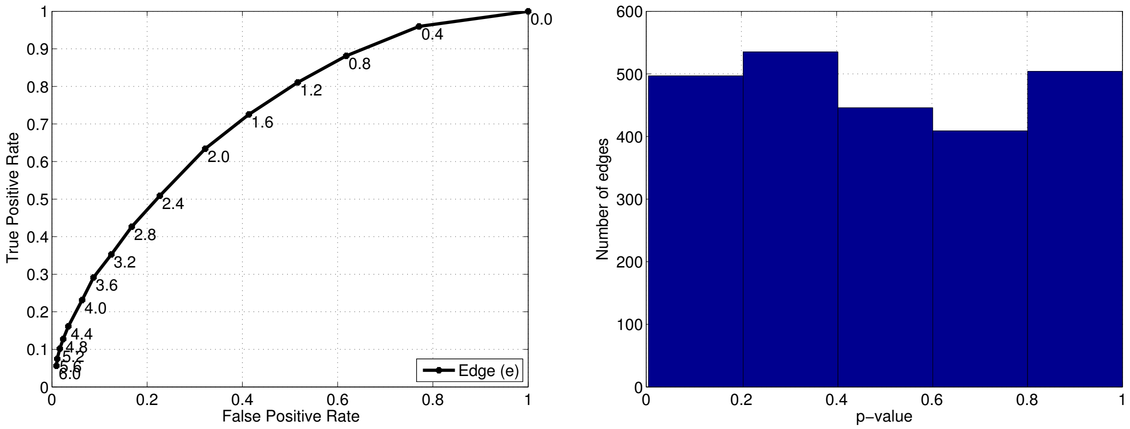

(a) (b)

Figure 4: This figure shows (a) the R.O.C. curve for the synthetic experiment (The critical value s^* of the test statistic is indicated.); and(b) the distribution of the p-values under the null hypothesis. The results obtained are for $N = 1000$ edges and a short simulation period $T = 8$

the functional categories based on the interactions of multiple genes which have the corresponding functional classification. Further, the strain damping allows the incorporation of multiple gene expression datasets which have been generated under slightly different conditions. In effect, we learn the dynamics of functional categories from multiple instances of short time series which are not i.i.d. but are strongly related through the functional classification. This reduces the variance in the results partly alleviating the problem of inference in short time series. The remainder of this section explores this in some detail.

The Markov transition probability Q_e of an edge $e \in E$ is a linear mixture of the Markov transition probabilities Q_h of the MIPS functional categories $\mathcal{C} = \{C_1, \ldots, C_H\}$, i.e.

$$Q_e = \sum_{h=1}^{H} \alpha_{e,h} Q_h \qquad \text{(D.15)}$$

where $\alpha_{e,h}$ denotes the influence of functional category C_h in the edge e such that $\sum_h \alpha_{e,h} = 1$ for all edges $e \in E$. Thus, there are multiple time series $\{w_e(t) : \alpha_{e,h} \geq 0\}$ for a functional category $h \in H$.

The functional categories were obtained by restricting to the functional category description to maximum depth 3, i.e., a functional category correspond to MIPS ID 10, 10.06, 31.4.29, etc. was included, whereas the

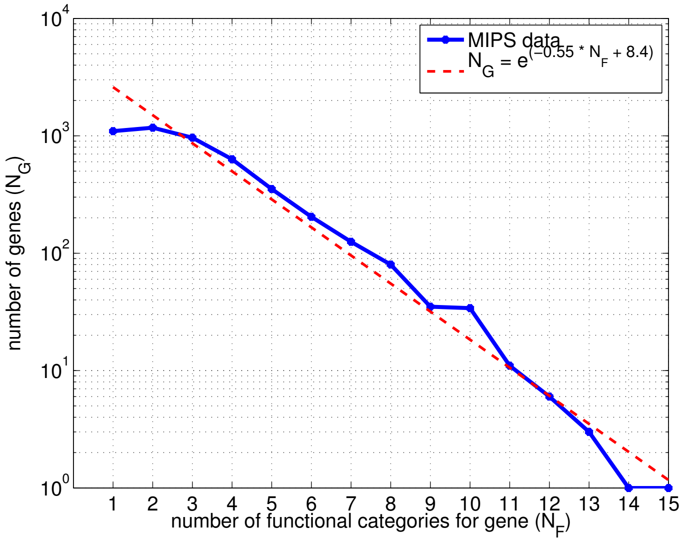

Figure 5: The distribution of the number of functional categories for 4715 genes over 260 MIPS functional categories used in Experiment 2. The average number of functional categories for a gene is 3.

functional categories corresponding to MIPS ID 10.4.25.1, 16.20.31.21, etc. were excluded.

Figure 5 shows the distribution of number of MIPS functional categories for a given gene, for 4715 genes with $H = 260$ possible categories (MIPS ID) from Experiment 2. The average number of functional categories for a gene is 3.

The presence of multiple edges corresponding to a functional category provides extra information. In order to elucidate, we present the following experiment: We generated a synthetic graph consisting of $N = 1000$ nodes and 5961 (randomly selected) edges $e \in E$ between them which can have interactions $\mathcal{W} = \{-2, -1, 0, 1, 2\}$. We use $H = 200$ functional categories which govern the behaviour of the network evolution through randomly selected mixing proportions $\alpha_{e,h}$ in (D.15) having distribution as Figure 5. The transition probabilities Q_h are generated randomly from two classes $H1 : tr(Q_h) \leq 0.5|\mathcal{W}|$ and $H0 : tr(Q_h) > 0.5|\mathcal{W}|$ $(P = 50\%)$. The experiment was run for $T = 8$ simulation period. We define change score $\tilde{s}_T(h)$ for the functional category C_h as $\tilde{s}_T(h) = \frac{\sum_{\{e \in E: \alpha_{e,h} \geq 0\}} \alpha_{e,h} s_T(e)}{\sum_{\{e \in E: \alpha_{e,h} \geq 0\}} \alpha_{e,h}}$.

Figure 2 in the main manuscript shows the comparison in ROC plots of

the edges $e \in E$ and functional categories C_h, based on the corresponding test statistics $s_T(e)$ and $\tilde{s}_T(h)$. We note that the multiple edges corresponding to a functional category improves the detection of significant functional categories even under short time periods.

E Discussion on NETGEM vs Naive HMM

This section presents a study of the naive HMM vs the NETGEM model. One can model the dynamics of the simple model defined by (1) and (2), using a simple HMM. The quantity to be estimated is the transition probability $\mathbf{Q}$. However, the exponential state space makes such an approach impractical. For the purpose of study, we explore the relation between our model and the simple HMM in the following two sections.

E.1 Evaluation of independent weights dynamics

We now discuss the relationship between the results obtained using the independent weights evolution assumption and the original problem.

Lemma E.1 *The independently evolving weights assumption gives a rank-1 tensor approximation Horn and Johnson [1990] to the global weight transition probability.*

For example, if the interaction network consists of two edges having transition probabilities $A = \{a_{ij}\}$ and B respectively, the approximation to the original matrix, $\hat{Q}$ is given as the Kronecker product

$$\hat{Q} = A \otimes B = \begin{bmatrix} a_{11}B & a_{12}B \\ a_{21}B & a_{22}B \end{bmatrix}$$

We note that if the weights indeed evolve independently of each other and we are given the edge transition probabilities Q_e, the overall system transition probability, $\mathbf{Q}$, is given as

$$\mathbf{Q} = Q_1 \otimes Q_2 \otimes \ldots \otimes Q_E$$

In general, if there are E edges with estimated transition probability matrices, $\hat{Q}_1, \ldots, \hat{Q}_E$, we obtain the approximation, $\hat{\mathbf{Q}}$

$$\hat{\mathbf{Q}} \simeq Q_1 \otimes Q_2 \otimes \ldots \otimes Q_E$$

We discuss the quality of approximation in case the original probability matrix Q is a higher order tensor elsewhere.

E.2 Number of parameters to be learnt

We note that the generative model, NETGEM, does an order reduction the number of parameters to be learnt. Specifically, if there are E edges in the network, with each edge taking on W denotes the number of possible interaction strengths, and H is the number of functional classes, the number of parameters to be learnt is reduced from $O(W^{2*E})$ to $O(HW^2 + HE)$. Typical values are $E \sim 6000$, $H \sim 260$ and $W \sim 5$.

E.3 Experimental comparison

We consider a model M which provides estimates $\{\hat{\mathbf{Q}}^M\}$ and $\{\hat{\mathbf{w}}_e^M(t)\}$ for the true transition probabilities $\mathbf{Q}$ and the actual interaction strengths $\{w_e(t)\}$ for the edges $e \in E$ for the time period $t \in T$. Then, a standard hidden Markov model attempts to estimate $\hat{\mathbf{Q}}^{HMM}$ directly. We compare our method (NETGEM) with a standard implementation of HMM[1].

We use two metrics to compare our results with respect to the standard hidden Markov model implementation over the large state space, namely,

1. *F1-score:* F1-score is the harmonic mean of the precision, P, and recall, R , i.e., $F1 = \frac{2PR}{(P+R)}$. In the context of our problem, the precision, P_M, and recall, R_M, for a model, M, are defined as

$$P_M = \frac{\sum_{e \in E} \sum_{t=1}^{T} \mathbf{1}_{\hat{w}_e^M(t)=1} \mathbf{1}_{w_e(t)=1}}{\sum_{e \in E} \sum_{t=1}^{T} \mathbf{1}_{\hat{w}_e^M(t)=1}}$$

$$R_M = \frac{\sum_{e \in E} \sum_{t=1}^{T} \mathbf{1}_{\hat{w}_e^M(t)=1} \mathbf{1}_{w_e(t)=1}}{\sum_{e \in E} \sum_{t=1}^{T} \mathbf{1}_{w_e(t)=1}}$$

   where $\mathbf{1}_{\{.\}}$ is the indicator function. Thus, F-score measures the accuracy of the predictions about strengths of interactions made by the model.

2. *Frobenius norm:* We measure the Frobenius norm between the estimated (by model M) transition probability, $\hat{\mathbf{Q}}^M$ and the true transition probability, $\mathbf{Q}$ used to generate the data, as

$$||\hat{\mathbf{Q}}^M - \mathbf{Q}||_F = \sqrt{\sum_{i,j} |q_{ij}^M - q_{ij}|^2}$$

[1] `http://people.cs.ubc.ca/~murphyk/Software/HMM/hmm.html`

where q_{ij} denotes the $(i,j)^{th}$ element of the matrix $\mathbf{Q}$. Thus, this measures the accuracy with which we can estimate the transition probability, Q.

Comparing NETGEM with HMM:

We choose a graph with $N = 7$ nodes (genes) with $E = 10$ (interactions) and $H = 10$ functional classes. The genes are randomly assigned classes such that each node is a member of $N_{av} = 1.5$ classes on average. A random transition probability matrix, Q_h, is generated for each interaction class, $h \in H$. This allows us to generate the edge transition probabilities Q_e; and consequently the overall transition probability $\mathbf{Q} = \otimes_{e \in E} Q_e$ where $\otimes$ denotes the Kronecker product. This allows us to generate the weight evolution $W_{1...,T}$ for time period T. The observed synthetic expression levels $X_{1...T}$ are generated using Gibbs Sampling based on the Boltzmann distribution.

We note that the small value of N allows direct estimation of the transition probability matrix $\hat{\mathbf{Q}}^{HMM}$ of size $|\mathcal{W}|^E \times |\mathcal{W}|^E$; and larger values of N can cause memory overflow. For example, if there are $E = 10$ edges; each of whose weights can take $W = 2$ values; the size of the transition probability matrix to be learnt is $2^{10} \times 2^{10}$.

The weights take possible values in $\mathcal{W} = \{-1, 1\}$, and the evolution characteristic, Q_e, for each weight is a mixture based on the interacting genes. We note that a value of $+1$ indicates that the genes are *activating* each other ; while -1 indicates the genes are *repressing* each other.

Figure 6 (a) shows the F1-scores for different simulation time-periods for 5 strains each missing at most 1 randomly selected node. A value of $\beta = 0.5$ was used for the experiment. We observe that NETGEM does significantly better than a naive HMM for small number of observation time-points. This happens to be the case for most genetic expression datasets.Similarly, Figure 6 (b) shows that NETGEM recovers the original transition probability matrix $\mathbf{Q}$ more accurately than HMM.

F Experiment 1 results

Figure 7 - Figure 14 show the time varying interaction strengths between the genes from Experiment 1. Each network is composed of all the genes from the eight clusters previously identified Farzadfard et al. [2010], and is shown for the eight time points for which gene expression was measured. The time stamps (in hours) are indicated below each network. The edge colors denote their interaction strength, which was classified as strong repressing

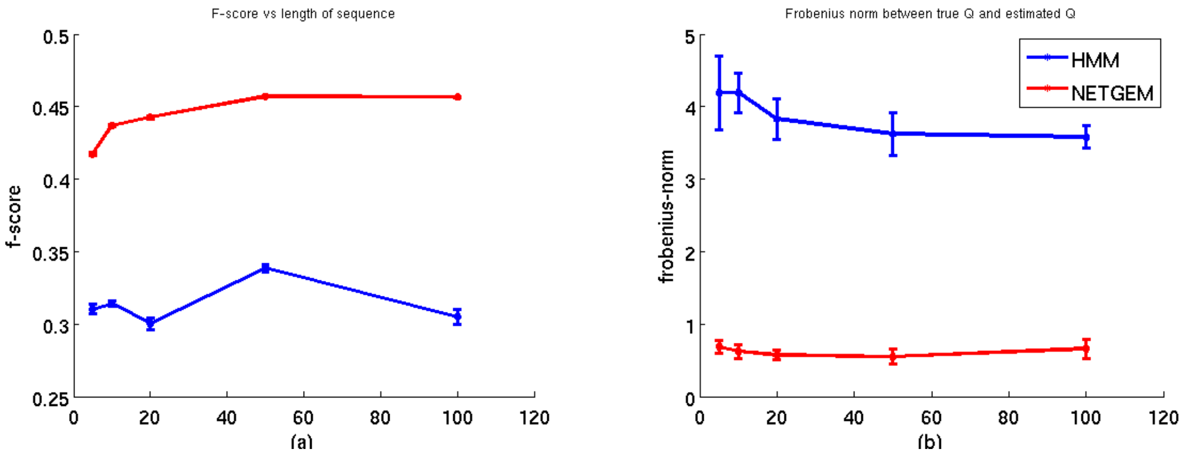

(a) (b)

Figure 6: This figure compares the results obtained using the generative model (NETGEM) with result of an standard HMM implementation with increasing number of observations (time points), T, available for 20 random trials using HMM (blue) and NETGEM (red) models. (a) F1-score for the estimated weight evolution *versus* the true weight evolution sequence (b) Frobenius norm of the difference between the true evolution characteristics for the system and the estimated characteristics.

(red), low repressing (pink), no effect (yellow), low inducing (light blue) and strong inducing (dark blue).

Figure 15 (a)-(t) shows the temporal evolution of the top 20 most changing (temporally) network interactions for the aerobic case.

G Experiment 2 results

Figures 16-21 present the complete interaction dynamics for Experiment 2 between genes in REF (a), MUT (b) and in both strains combined, using the damping model (c) at various time points. All the genes identified to be significantly changed Cipollina et al. [2008] were combined into one network. The color of the edges in the network indicates the interaction strength, which was classified as strong repressing (red), low repressing (pink), no effect (yellow), low inducing (light blue) and strong inducing (dark blue). The time stamps (in minutes) are indicated below each network.

Figure 22 (a)-(t) shows the temporal evolution for the top 20 most dynamic interactions using the damping model for joint analysis of the REF and MUT strains. Figure 23 (a) shows the statistics corresponding to the inferred interaction dynamics for Experiment 2. Figure 23 (b) shows the variation of damping coefficients due to gene knockout in the perturbed strain.

References

C. Cipollina, J. van den Brink, P. Daran-Lapujade, J. T. Pronk, D. Porro, and J. H. de Wind. Saccharomyces cerevisiae sfp1: at the crossroads of central metabolism and ribosome biogenesis. *Microbiology*, 154, 2008.

Jason Ernst and Ziv B. Joseph. Stem: a tool for the analysis of short time series gene expression data. *BMC Bioinformatics*, 7(1), 2006. doi: 10.1186/1471-2105-7-191. URL `http://dx.doi.org/10.1186/1471-2105-7-191`.

Jason Ernst, Gerard J. Nau, and Ziv Bar-Joseph. Clustering short time series gene expression data. In *ISMB (Supplement of Bioinformatics)*, pages 159–168, 2005.

F. Farzadfard, M. L. Nielsen, J. Nielsen, and G. N. Vemuri. Metabolic and transcriptional dynamics during the transition from carbon limitation to nitrogen limitation in saccharomyces cerevisiae. *BMC Genomics (in review)*, X, 2010.

Roger A. Horn and Charles R. Johnson. *Matrix Analysis*. Cambridge University Press, February 1990. ISBN 0521386322. URL http://www.amazon.com/exec/obidos/redirect?tag=citeulike07-20&path=ASIN/0521386322.

R.M. Neal and G.E. Hinton. A view of the EM algorithm that justifies incremental, sparse, and other variants. *Learning in graphical models*, 89: 355–368, 1998.

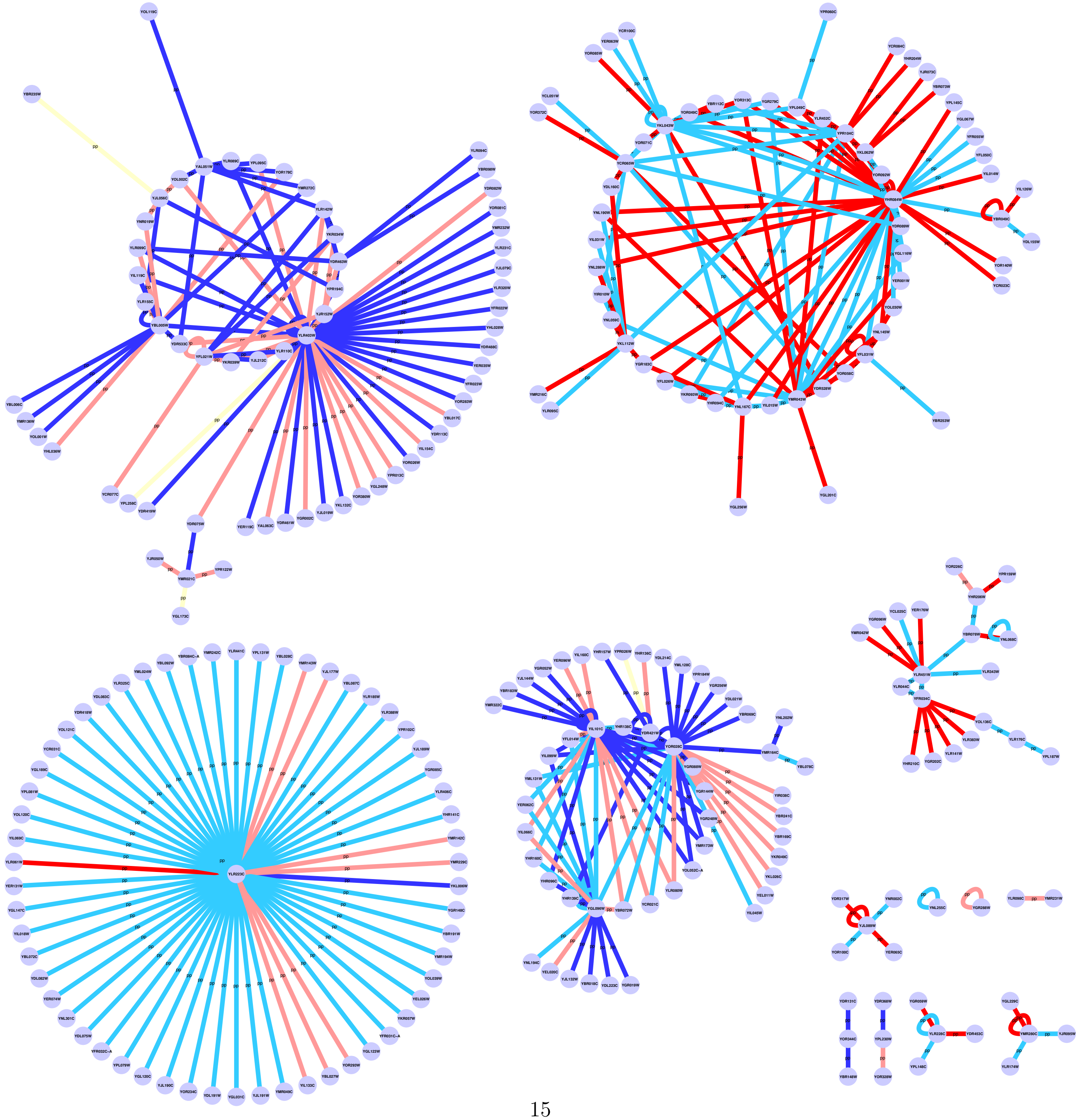

Figure 7: Experiment 1 network interactions at t=0 hr

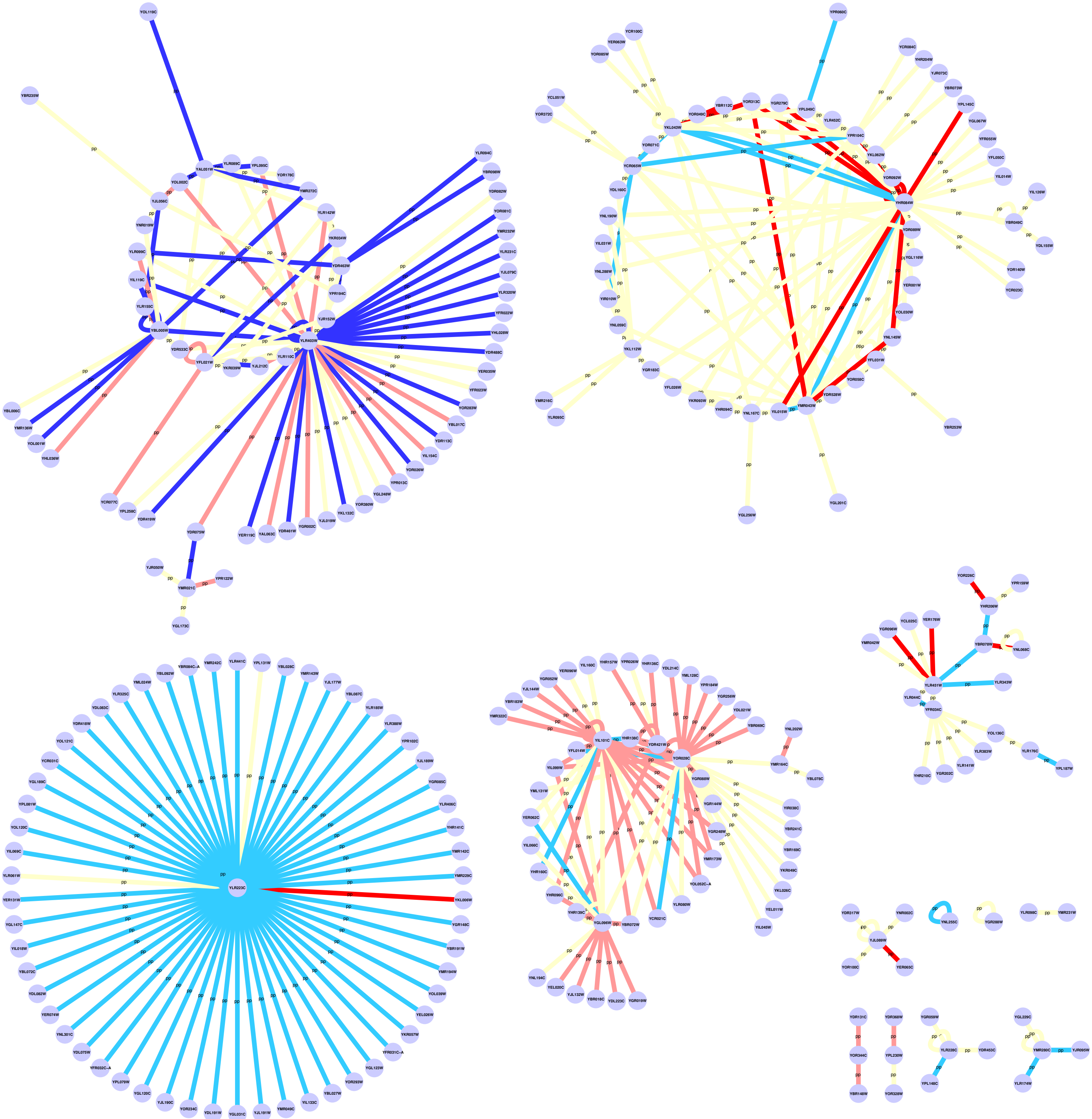

Figure 8: Experiment 1 network interactions at t=4.1 hr

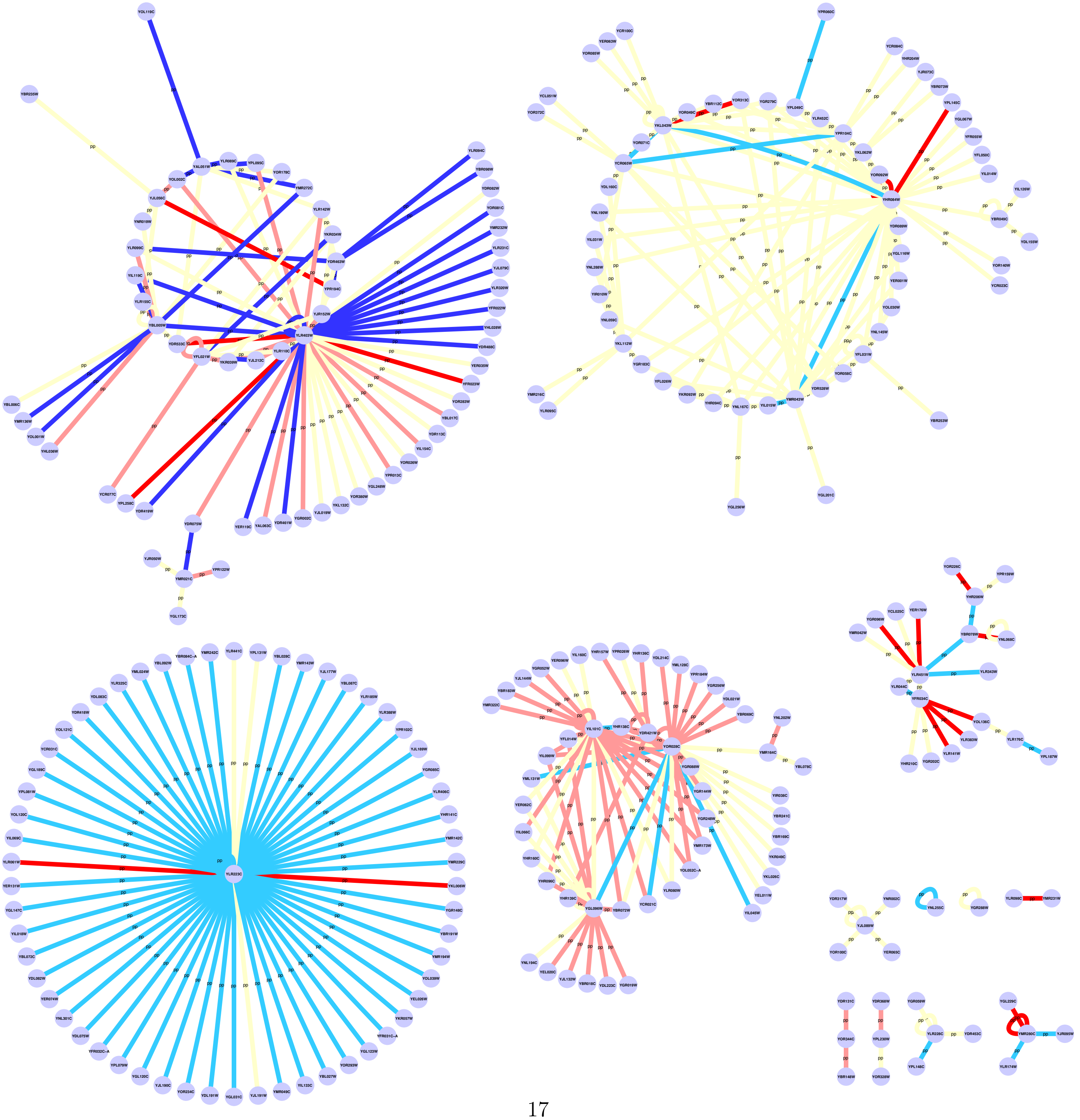

Figure 9: Experiment 1 network interactions at t=6.8 hr

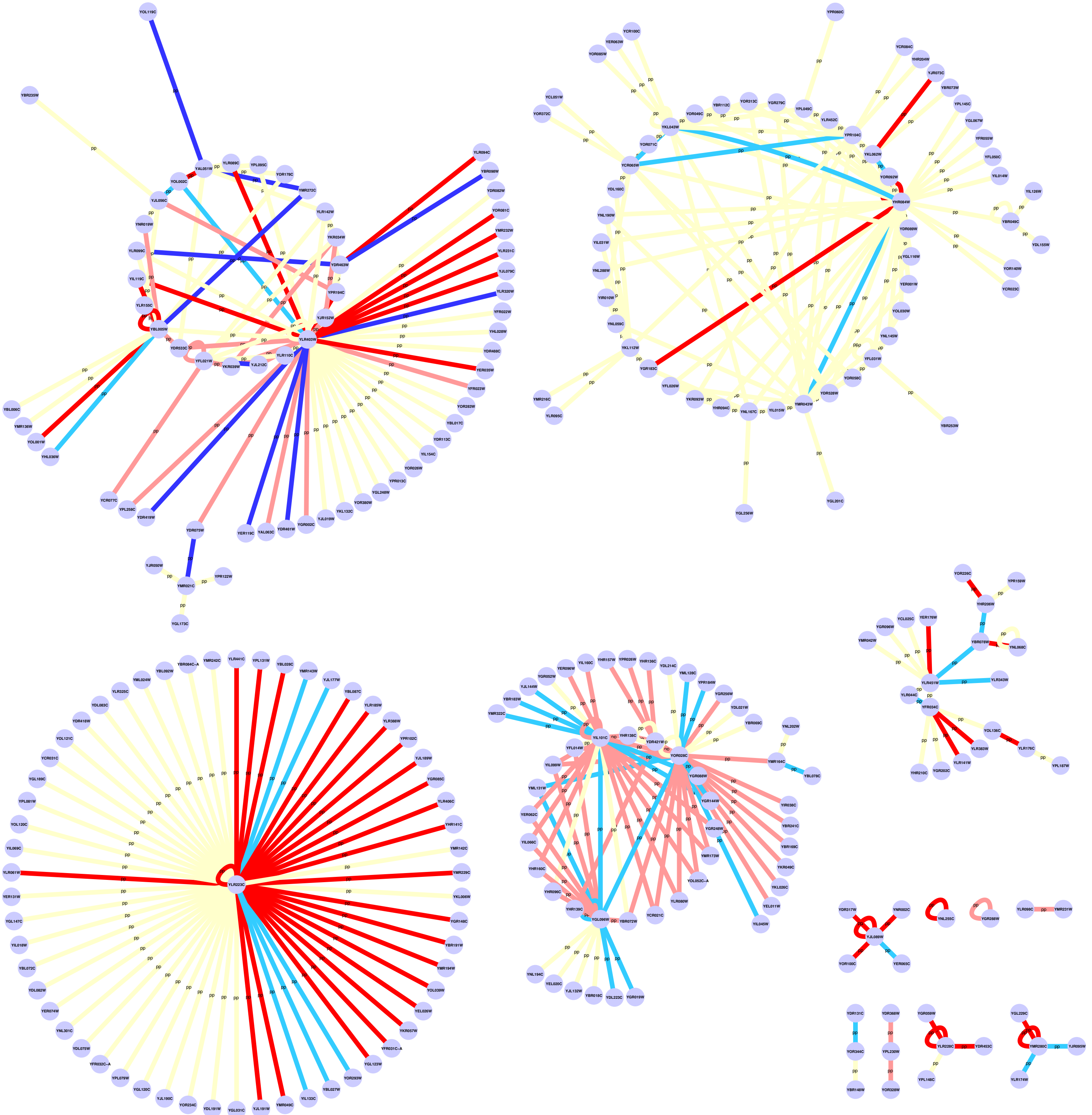

Figure 10: Experiment 1 network interactions at t=9.1 hr

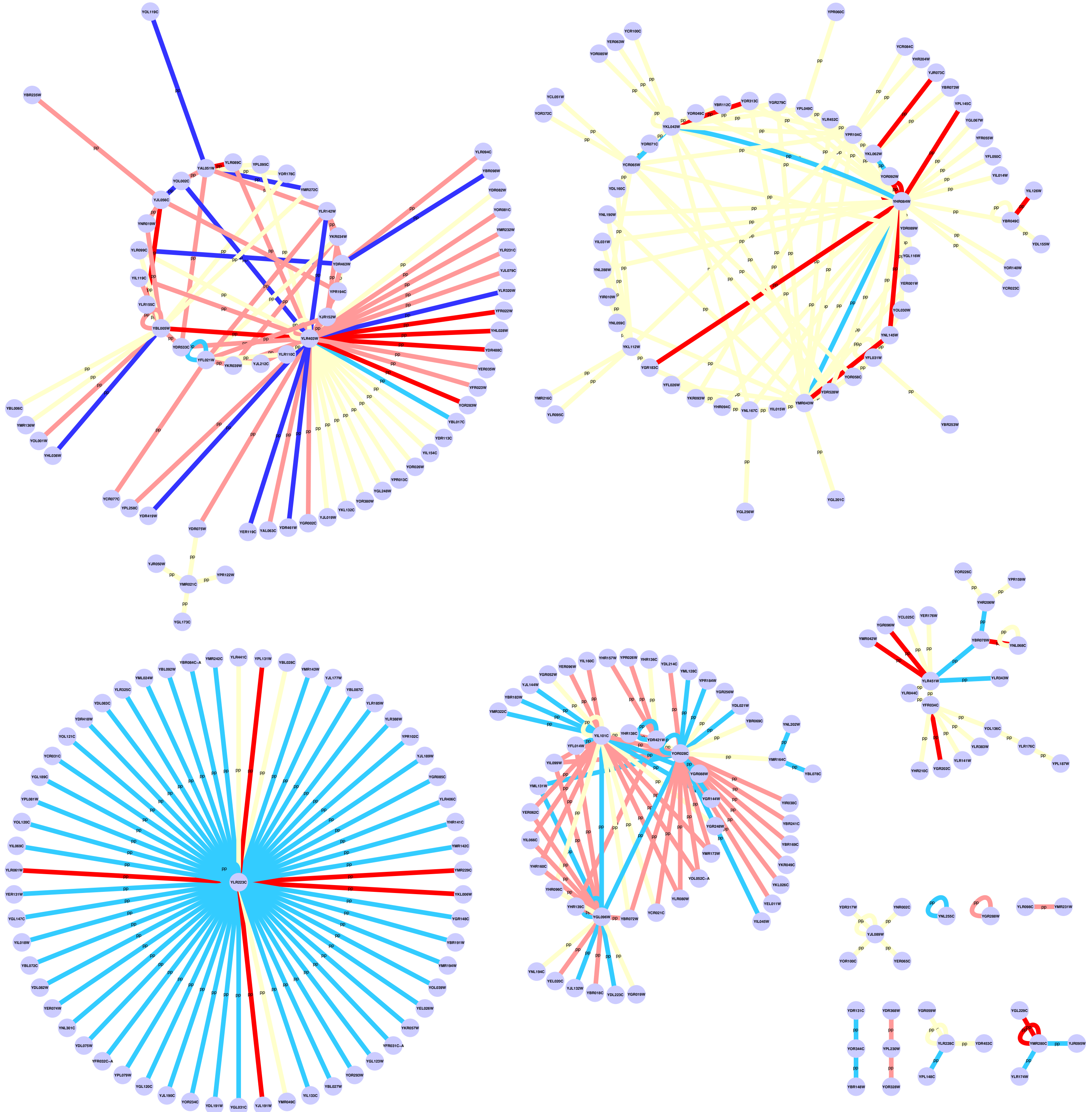

Figure 11: Experiment 1 network interactions at t=14.5 hr

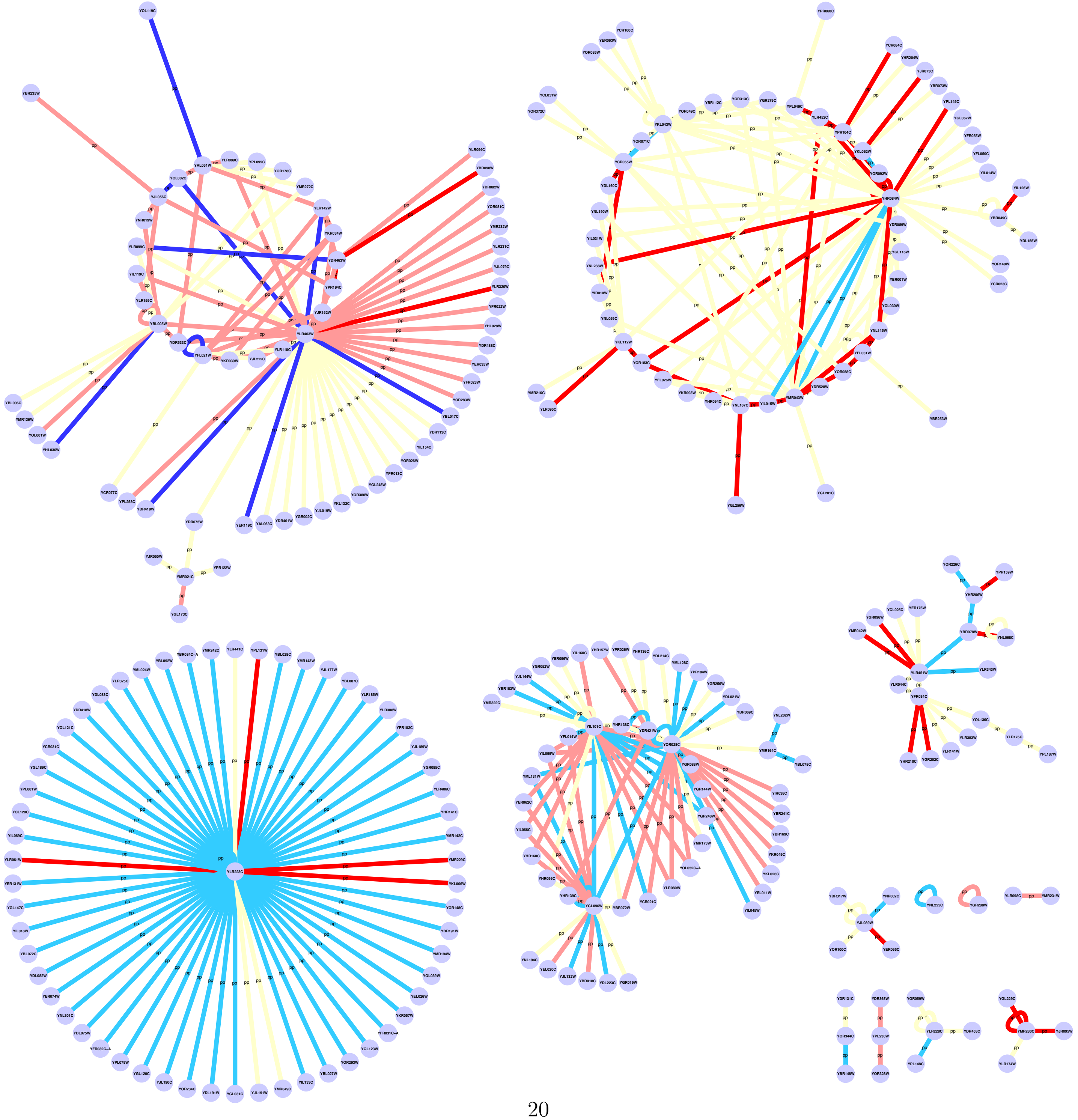

Figure 12: Experiment 1 network interactions at t=27.3 hr

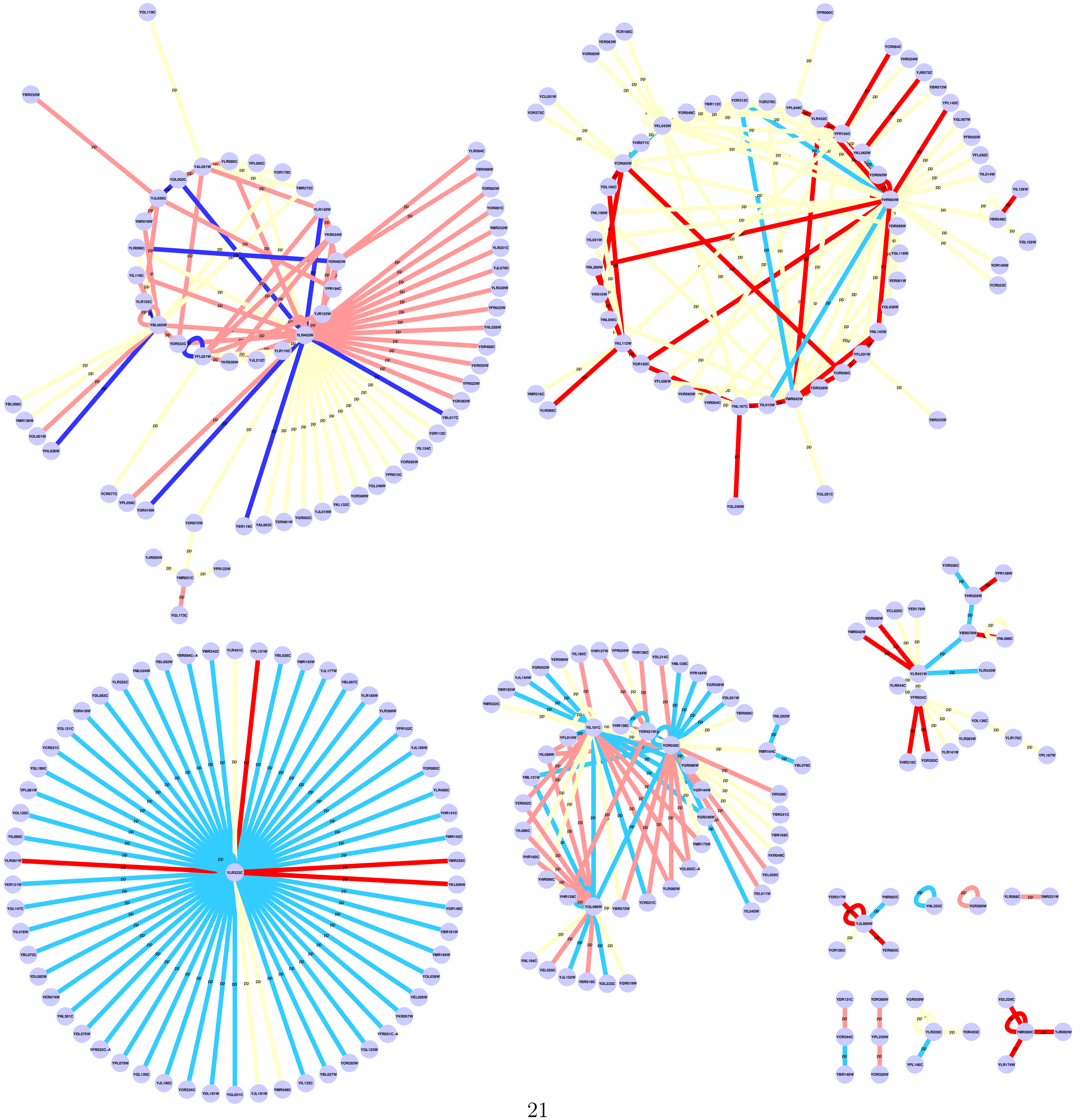

Figure 13: Experiment 1 network interactions at t=42.7 hr

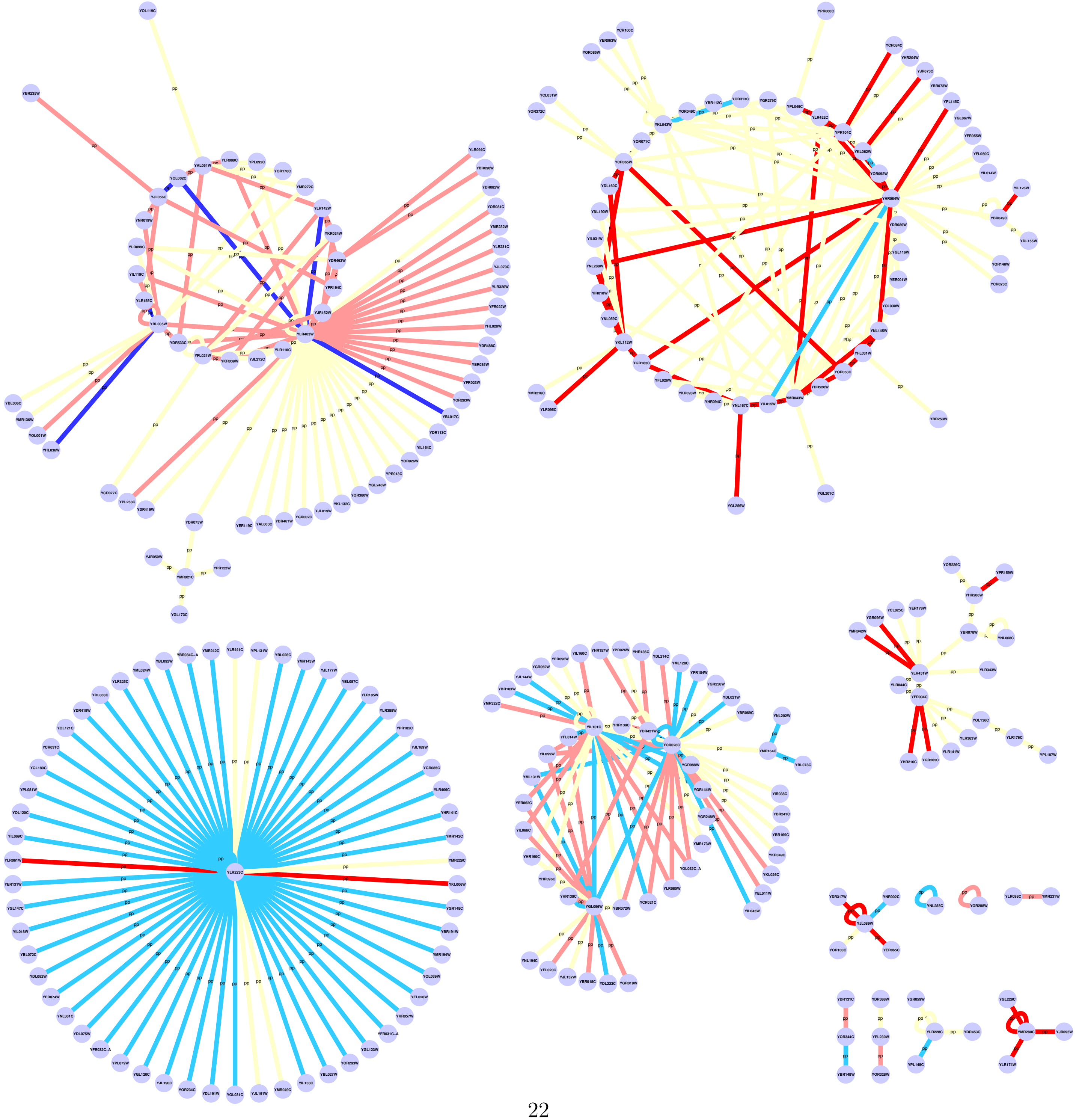

Figure 14: Experiment 1 network interactions at t=53.9 hr

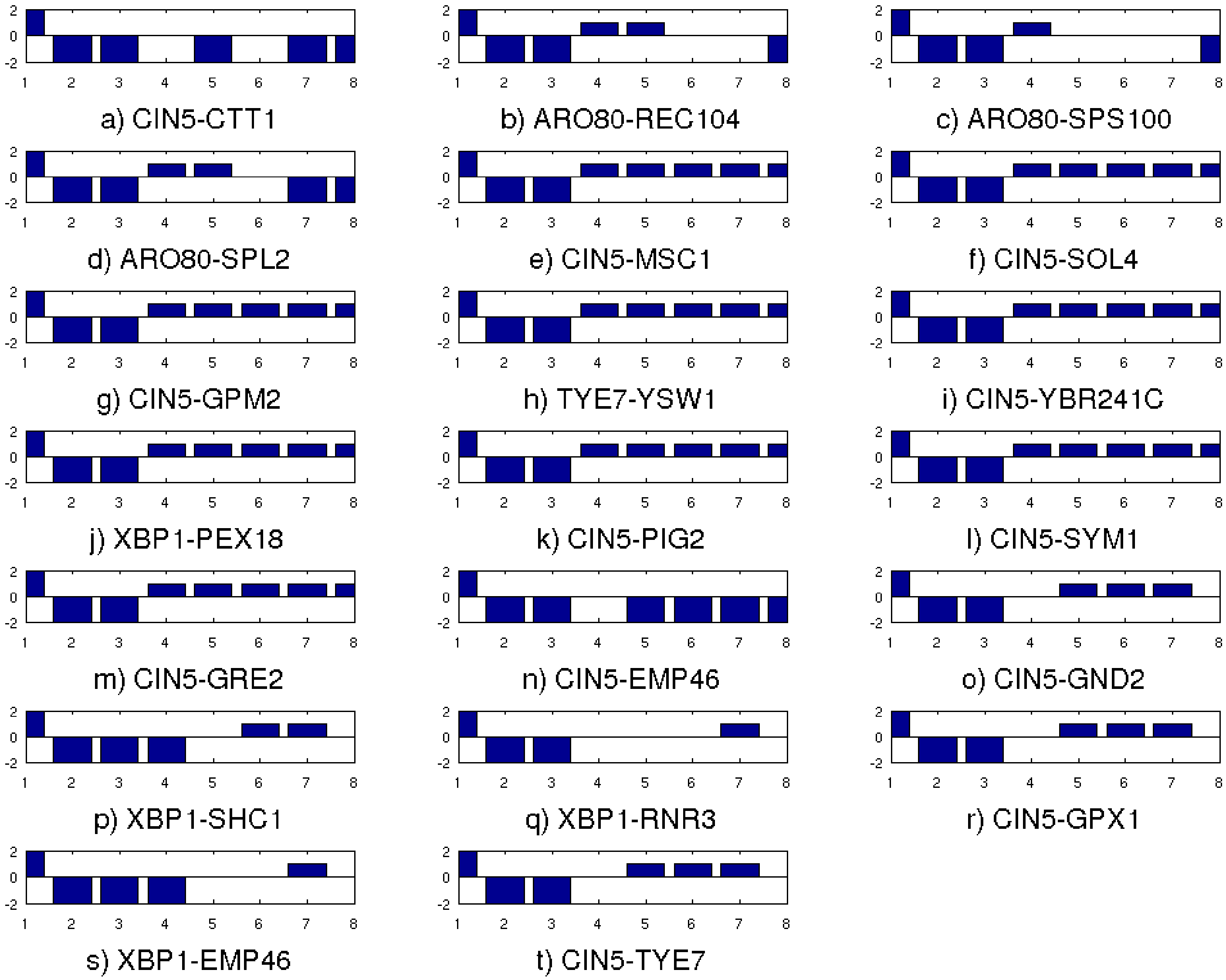

Figure 15: This figure shows the top 20 most dynamic interactions for Experiment 1 in the aerobic setting.

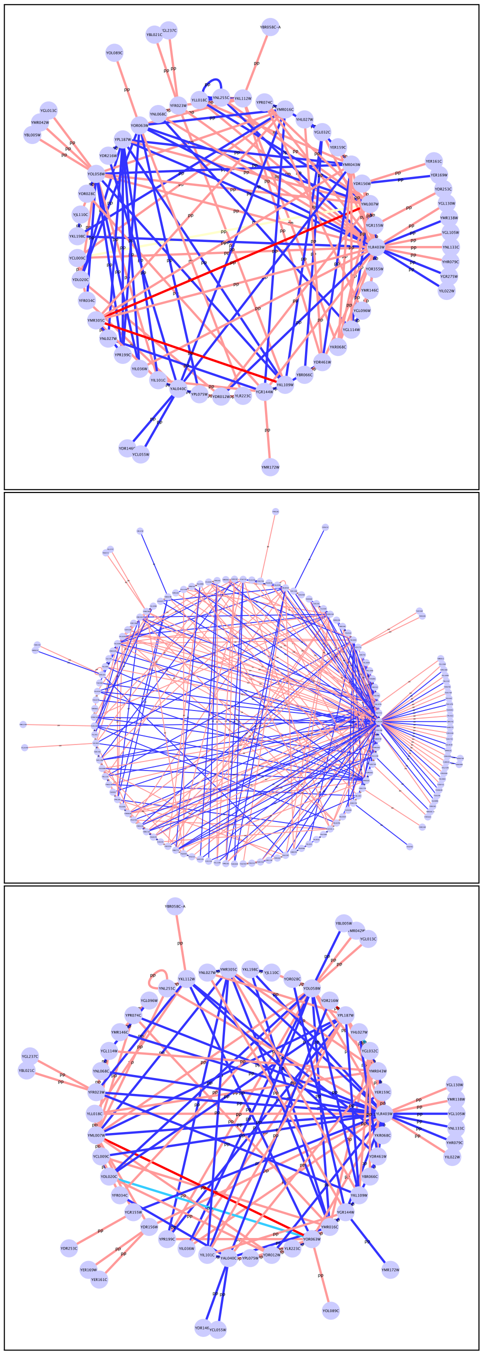

a.

b.

c.

Figure 16: Experiment 2 network interactions at $t = 0$ min

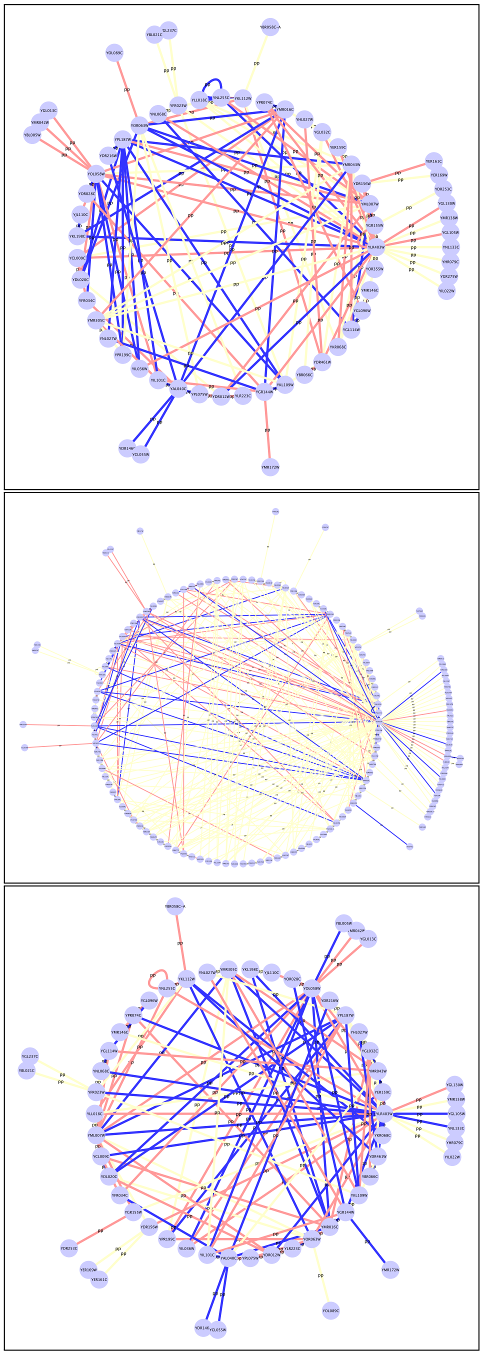

a.

b.

c.

Figure 17: Experiment 2 network interactions at $t = 5$ min

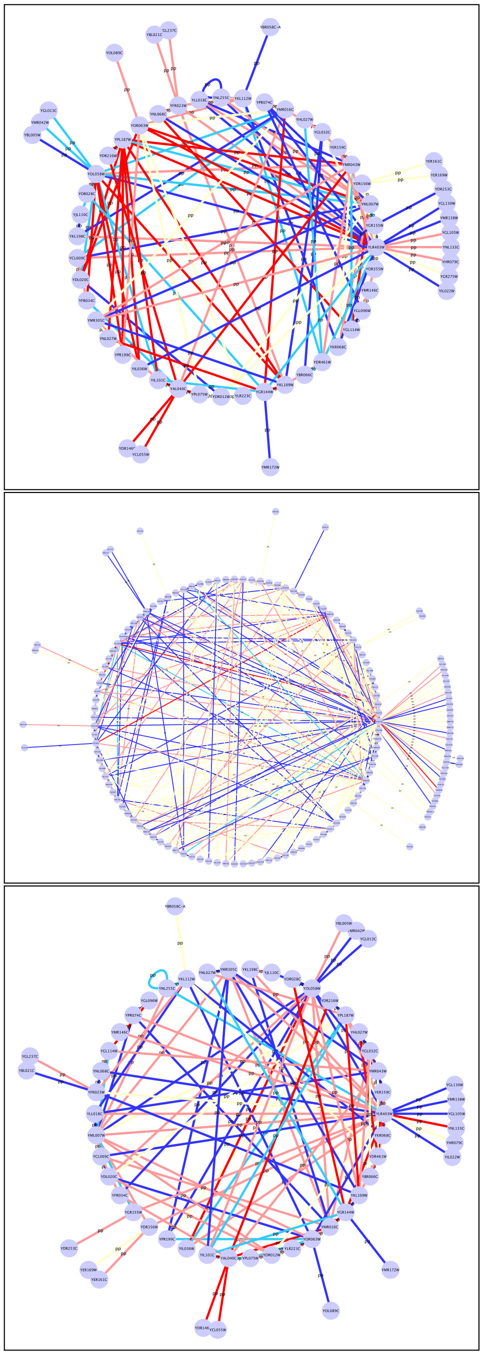

a.

b.

c.

Figure 18: Experiment 2 network interactions at $t = 10$ min

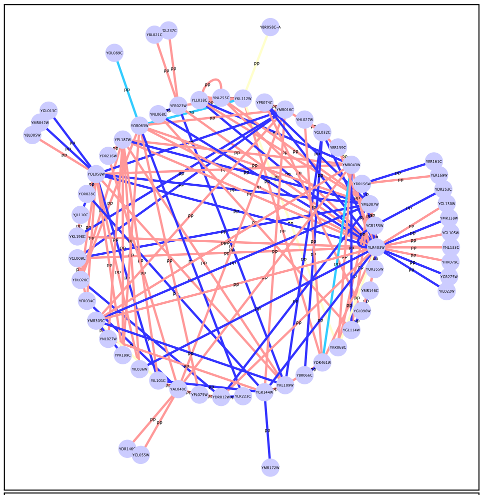

a.

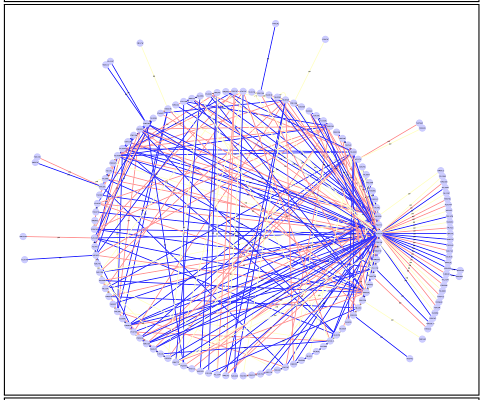

b.

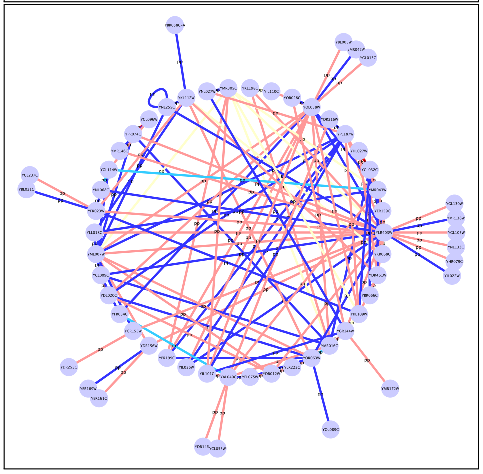

c.

Figure 19: Experiment 2 network interactions at $t = 30$ min

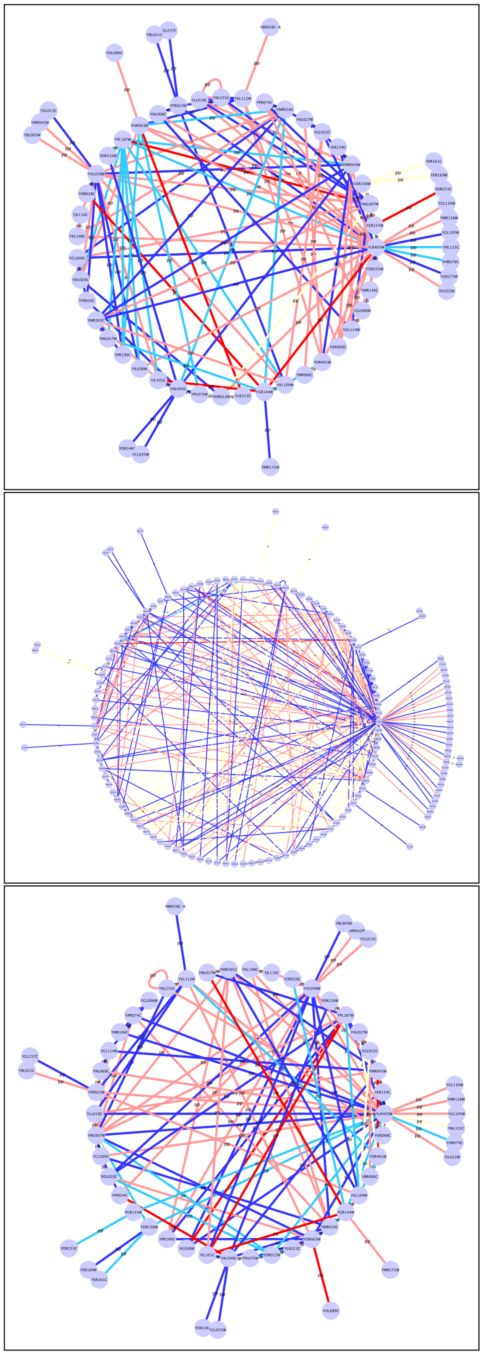

a.

b.

c.

Figure 20: Experiment 2 network interactions at $t = 60$ min

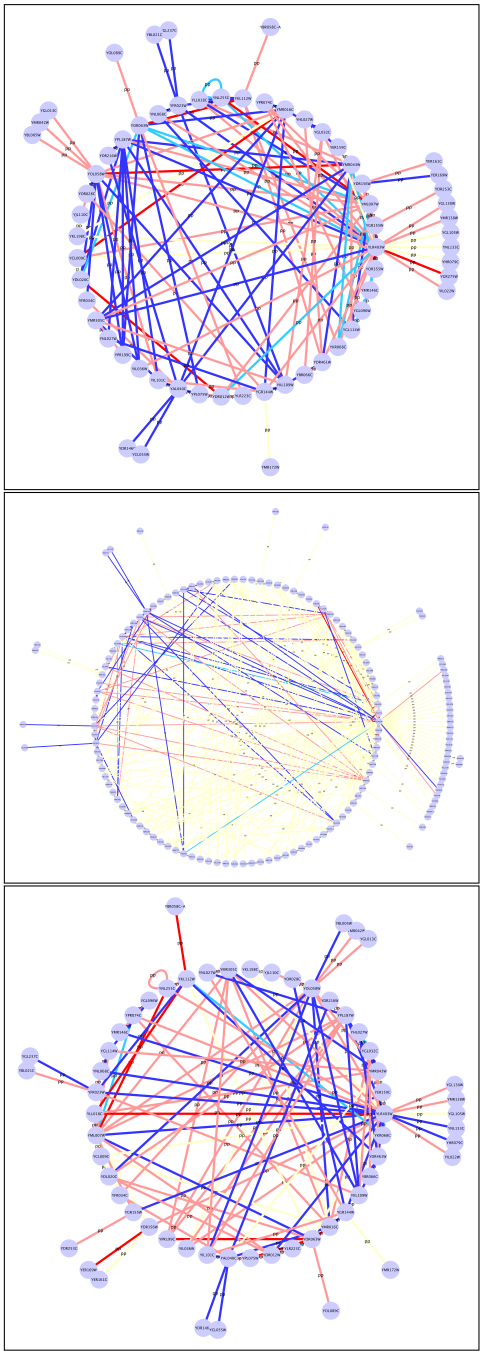

a.

b.

c.

Figure 21: Experiment 2 network interactions at $t = 120$ min

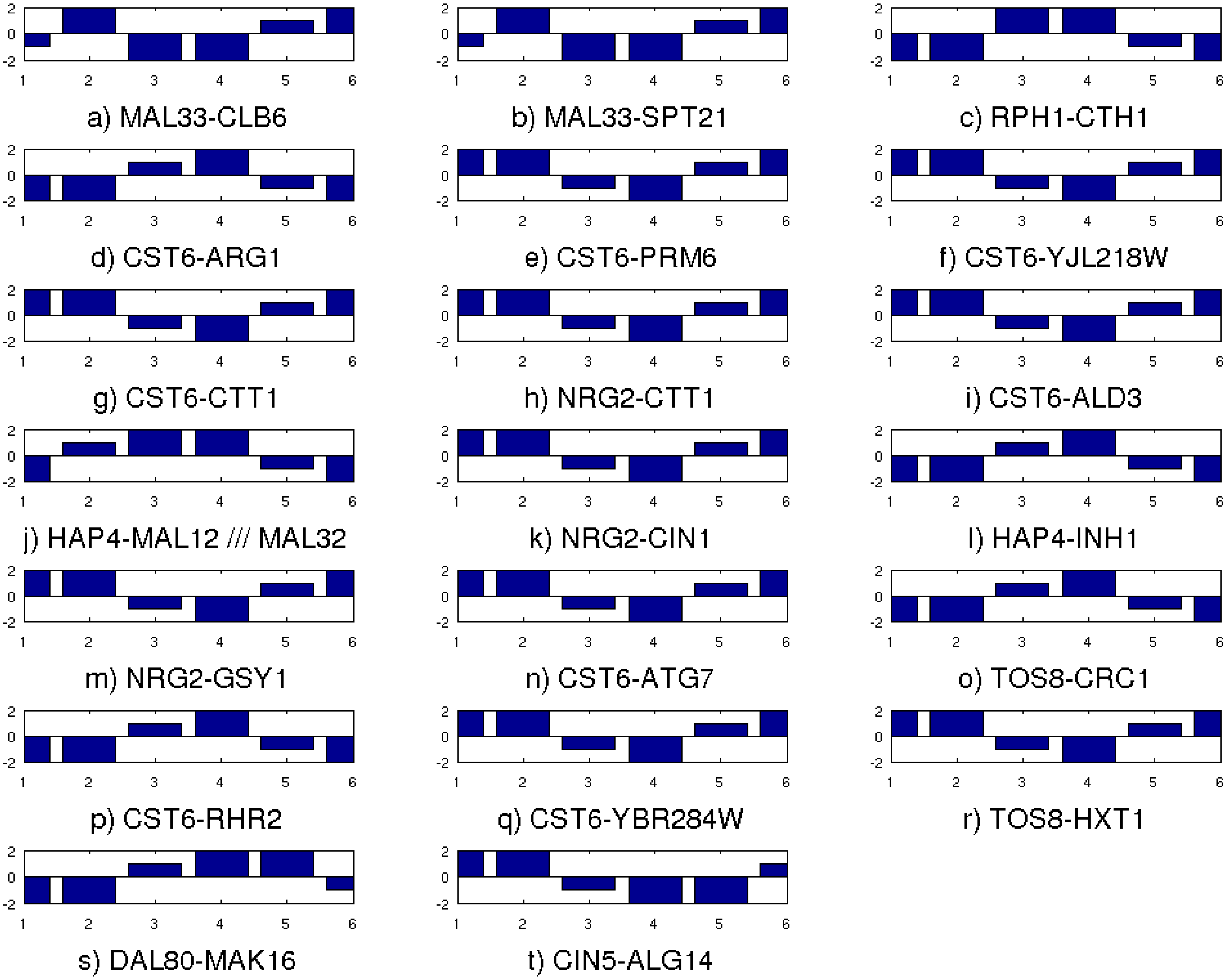

Figure 22: This figure shows the top 20 most time-varying edges for Experiment 2 using the damping model for joint analysis of the REF and MUT strains.

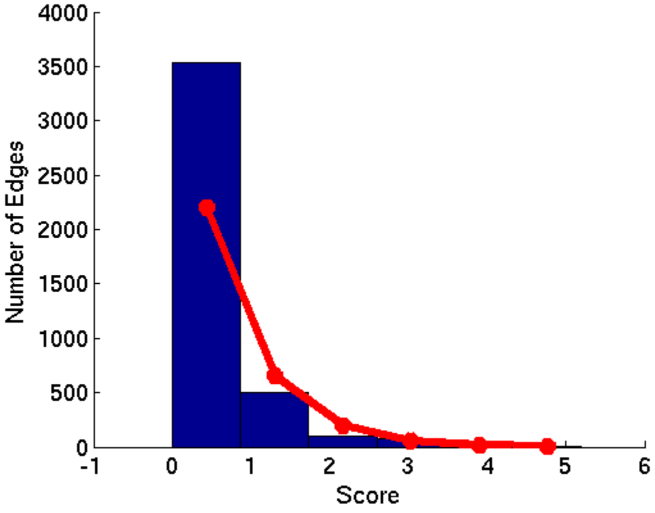

a.

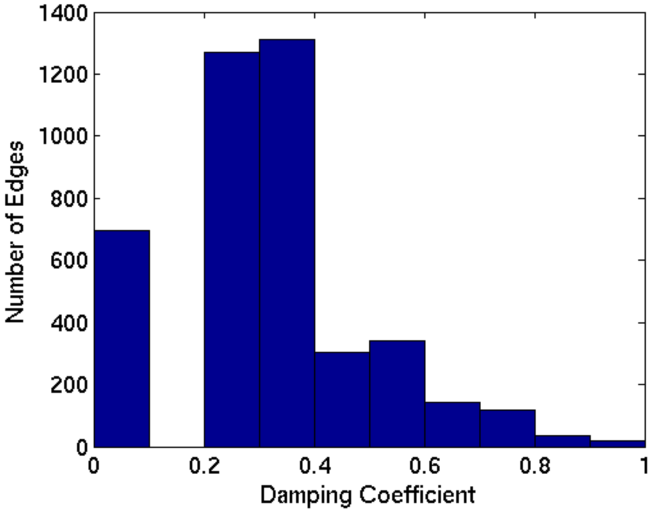

b.

Figure 23: This figure presents histograms characterizing the change in the interaction strengths over all edges when the inference is done over JOINT (both strain) (a). The line in red shows the exponential distribution with parameter chosen that it best fits the observed (histogram) data for the change score. We also show the histogram of the damping coefficients for the edges in the perturbed strain in Experiment 2 (b). It is important to note that the damping coefficients are dependent on the network topology.
